# *ASAR15*, A *cis*-Acting Locus that Controls Chromosome-Wide Replication Timing and Stability of Human Chromosome 15

**DOI:** 10.1371/journal.pgen.1004923

**Published:** 2015-01-08

**Authors:** Nathan Donley, Leslie Smith, Mathew J. Thayer

**Affiliations:** Department of Biochemistry and Molecular Biology, Oregon Health & Science University, Portland, Oregon, United States of America; University of Pennsylvania, United States of America

## Abstract

DNA replication initiates at multiple sites along each mammalian chromosome at different times during each S phase, following a temporal replication program. We have used a Cre/loxP-based strategy to identify *cis*-acting elements that control this replication-timing program on individual human chromosomes. In this report, we show that rearrangements at a complex locus at chromosome 15q24.3 result in delayed replication and structural instability of human chromosome 15. Characterization of this locus identified long, RNA transcripts that are retained in the nucleus and form a “cloud” on one homolog of chromosome 15. We also found that this locus displays asynchronous replication that is coordinated with other random monoallelic genes on chromosome 15. We have named this locus ASynchronous replication and Autosomal RNA on chromosome 15, or *ASAR15*. Previously, we found that disruption of the *ASAR6* lincRNA gene results in delayed replication, delayed mitotic condensation and structural instability of human chromosome 6. Previous studies in the mouse found that deletion of the *Xist* gene, from the X chromosome in adult somatic cells, results in a delayed replication and instability phenotype that is indistinguishable from the phenotype caused by disruption of either *ASAR6* or *ASAR15*. In addition, delayed replication and chromosome instability were detected following structural rearrangement of many different human or mouse chromosomes. These observations suggest that all mammalian chromosomes contain similar *cis*-acting loci. Thus, under this scenario, all mammalian chromosomes contain four distinct types of essential cis-acting elements: origins, telomeres, centromeres and “inactivation/stability centers”, all functioning to promote proper replication, segregation and structural stability of each chromosome.

## Author Summary

Mammalian cells replicate their DNA along each chromosome during a precise temporal replication program. In this report, we used a novel “chromosome-engineering” strategy to identify a DNA element that controls this replication-timing program of human chromosome 15. Characterization of this element indicated that it encodes large non-protein-coding RNAs that are retained in the nucleus and form a “cloud” on one copy of chromosome 15. Previously, we found that structural rearrangements of a similar element on human chromosome 6 causes delayed replication and structural instability of chromosome 6. Mammalian chromosomes are known to contain three distinct types of essential DNA elements that promote proper chromosome function. Thus, every chromosome contains: 1) origins of replication, which are responsible for proper initiation of DNA synthesis; 2) centromeres, which are responsible for proper chromosome separation during cell division; and 3) telomeres, which are responsible for replication and protection of the ends of linear chromosomes. Our work supports a model in which all mammalian chromosomes contain a fourth type of essential DNA element, the “inactivation/stability center”, which is responsible for proper DNA replication timing and structural stability of each chromosome.

## Introduction

Morphological differences between chromosomes residing within the same cell were first observed in mammalian cells nearly fifty years ago (reviewed in [1]). The phenomenon of “chromosome pulverization” was first described in human cells, where infection with the measles virus causes a severe fragmentation that gives the appearance that the chromosomes have been “pulverized” [2], [3]. Subsequently, abnormal chromosome condensation was shown to occur when viruses cause mitotic cells to fuse with interphase cells [4]. This premature chromosome condensation (PCC) phenotype was found to affect one of the two complete sets of chromosomes present in heterokaryons when mitotic cells are fused with other cells in different phases of the cell cycle. The PCC is most dramatic when S-phase cells were fused to mitotic cells, suggesting that condensation of partially replicated chromosomes causes the pulverized appearance [5]. In contrast, a morphologically similar abnormal chromosome condensation phenotype was observed on one or a few chromosomes during mitoses of cancer cell lines [6]–[8], primary tumor cells [7], and in cells exposed to mitotic spindle poisons [9]–[11] or DNA damage [12]–[15]. Moreover, we found that certain tumor-derived rearranged chromosomes exhibit a delay in replication timing (DRT), which is characterized by a>2 hour delay in the initiation and completion of DNA synthesis along the entire length of the chromosome [7]. Chromosomes with DRT also display a delay in mitotic chromosome condensation (DMC), which is characterized by an under-condensed appearance and a concomitant delay in phosphorylation of serine 10 of histone H3 [7], [16]. DRT/DMC chromosomes were also detected in 5 of 7 tumor cell lines and in 5 of 13 primary tumor samples, indicating that DRT/DMC is common in human cancer cells *in vitro* and *in vivo* [7]. Subsequently, we found that chromosomes with DRT/DMC are present in as many as 25% of cells surviving exposure to ionizing radiation (IR), and that IR induced DRT/DMC on mouse chromosomes can persist for up to 2 years *in vivo* [15]. Importantly, DRT/DMC occurred predominantly on chromosome translocations, estimated to be ∼5% of all translocations induced by IR, indicating that structural rearrangement is responsible for the DRT/DMC phenotype [15].

We have developed a chromosome engineering system that allows for the systematic analysis of chromosomes with DRT/DMC [15]–[19]. Our strategy is based on the Cre/loxP system to generate precise chromosomal rearrangements. Using this system we previously identified five balanced translocations, affecting eight different autosomes, each displaying DRT/DMC on at least one of the two derivative chromosomes [17]. Subsequently, we found that translocations or deletions at a discrete *cis*-acting locus on human chromosome 6 result in DRT/DMC. Characterization of this locus identified a large intergenic non-coding RNA (lincRNA) gene, which we named ASynchronous replication and Autosomal RNA on chromosome 6, or *ASAR6* [19]. The *ASAR6* gene displays random monoallelic expression, and as the name implies displays asynchronous replication that is coordinated with other monoallelic genes on chromosome 6 [18], [19]. The ASAR6 RNA is a long (>200 kb), non-spliced, non-polyadenylated, RNA Polymerase II product that is retained within the nucleus at or near the site of transcription [18], [19]. In addition, disruption of the expressed allele of *ASAR6* results in transcriptional activation of previously silent alleles of other monoallelic genes on chromosome 6 [19].

In the current report we show the characterization of a *cis*-acting locus present at the breakpoint of a second balanced translocation identified in our original screen for DRT/DMC chromosomes [17]. In this report, we found that deletions, or inversions at a complex locus on chromosome 15q24.3 cause DRT/DMC and structural instability of human chromosome 15. This locus encodes the large protein-coding gene *SCAPER*, the micro RNA gene *MIR3713*, and long RNA transcripts that are retained within the nucleus and form a “cloud” on one homolog of chromosome 15. Because this locus also displays asynchronous replication that is coordinated with other random monoallelic genes on chromosome 15, we have named this locus ASynchronous replication and Autosomal RNA on chromosome 15, or *ASAR15*.

## Results

### DRT/DMC on a balanced t (15;16)

**Figure 1:**
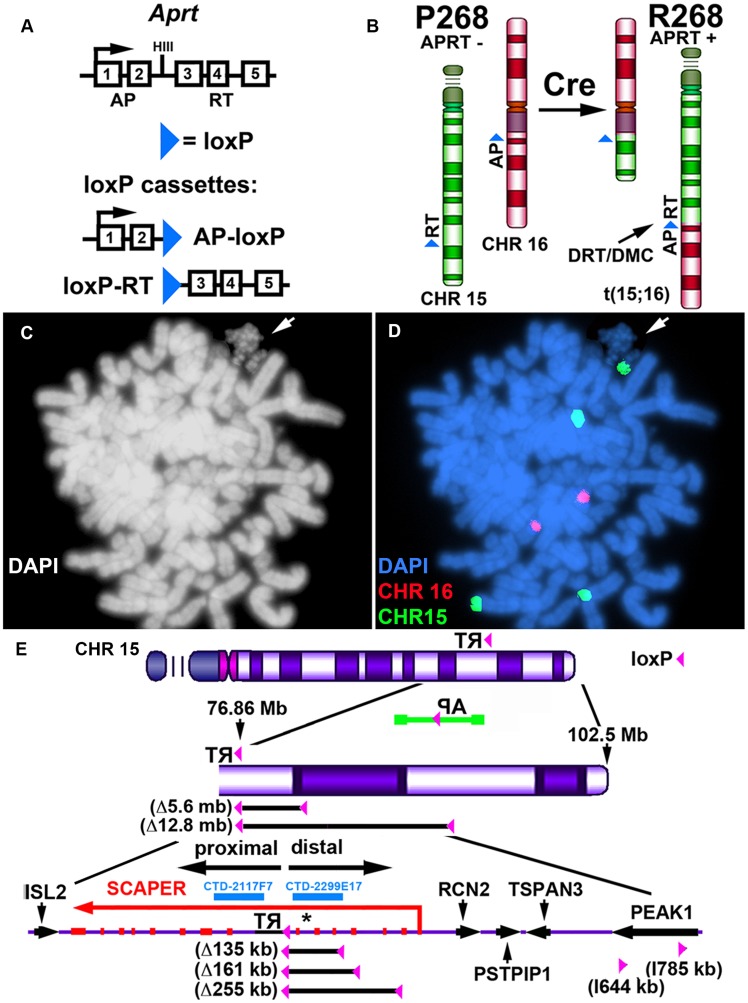
DRT/DMC on an engineered t (15;16). A) Diagram of the Aprt-loxP cassettes, and the genomic organization of the mouse *Aprt* gene is shown. The 5′ portion of the Aprt gene was separated from the 3′ portion to generate the AP-loxP and loxP-RT cassettes. Each cassette contains a loxP site in the second intron of the Aprt gene, and each cassette was cloned separately into Lentiviral vectors. B) Illustration of the original loxP cassette integration sites in chromosomes 15 (green) and 16 (red) in P268 cells, and the balanced translocation, t (15;16), generated in R268 cells (see [17]). C and D) DMC on the chromosome 15 derivative of the t (15;16). R268 cells were harvested for mitotic cells, dropped on slides and processed for DNA FISH using chromosome 15 and 16 centromeric probes [CHR15 (green) and CHR16 (red)]. E) Diagram of chromosome 15 showing the orientation and integration site (∼76.86 mb) of the original loxP-3′RT cassette in P268 cells. P268 cells were infected with Lentiviral vectors containing either the AP-loxP or loxP-RT cassettes, and 18 pools of 5,000–10,000 infected clones isolated for each Lentivirus. The structure of the AP-loxP Lentivirus (in the opposite orientation; green), the extent of 5 distal deletions (Δ135 kb, Δ161 kb, Δ255 kb, Δ5.6 mb and Δ12.8 mb) and 2 inversions (I644 kb, and I785 kb), BACs (CTD-2117F7 and CTD-2299E17), and the protein-coding genes *ISL2*, *SCAPER, RCN2, PSTPIP1, TSPAN3* and *PEAK1* are indicated. The approximate location of the micro RNA gene *MIR3713* is shown with an asterisk.

Our chromosome engineering strategy is based on the Cre/loxP site-specific recombinase system to generate chromosomal rearrangements (reviewed in [20]). Because Cre/loxP is relatively inefficient at mediating inter-chromosomal events, we are using reconstitution of a selectable marker to isolate the cells that undergo Cre-mediated recombination (see Fig. 1A). Thus, following Cre-mediated recombination and selection for reconstitution of the *Aprt* gene, reciprocal exchanges are generated in recombinant clones (R-lines) of Aprt+ cells [17]. Fig. 1B illustrates the balanced translocation generated by Cre expression in the parental P268 cell line, which was identified during our original screen for DRT/DMC [17]. The balanced translocation generated in the resulting recombinant (R268) cells involves the long arms of chromosomes 15 and 16, and the chromosome 15 derivative displays the DRT/DMC phenotype (Fig. 1C and D).

### Engineering deletions in chromosomes 15 and 16

Using our chromosome engineering system we previously found that Cre/loxP-mediated translocations and subsequently deletions affecting the *ASAR6* gene result in DRT/DMC on chromosome 6 [17]–[19]. Using a similar strategy we next determined if deletions in either chromosome 15 or 16 could cause DRT/DMC in the P268 cell line. For this analysis we used a random Lentiviral integration approach to introduce new loxP cassettes into P268 cells. The rationale behind this set of experiments is based on previous studies showing that intra-chromosomal Cre events are more efficient than inter-chromosomal Cre events [21]. Thus, expression of Cre in pools of Lentiviral infected cells reconstitutes *Aprt* at a higher efficiency in cells containing Lentiviral integrations near the original loxP cassettes, and depending on the orientation of the newly integrated cassettes either deletions or inversions are generated following Cre transient expression [19]. Using this random integration approach we generated 14 different deletions in chromosome 15, and 9 different deletions in chromosome 16 (in Δ268 clones). Note that this approach resulted in nested deletions anchored at the original loxP cassettes in both chromosomes 15 and 16 (see Fig. 1E and Table 1). In addition, this approach allowed us to isolate multiple independent clones containing the same deletions. Thus, for the majority of the deletions characterized in this study we analyzed multiple independent clones representing the same Cre/loxP-mediated deletions (see S1 Table).

**Table 1:** Cre/loxP-mediated chromosome 15 rearrangements.

Chromosome 15 deletions		Deletion Size (base pairs)		DRT/DMC#	Instability^q^
Clone Name	Integration Site	Proximal	Distal	Clones +	Clones +
Δ268F-6a	cen-58,733,512	>18,125,231		0(1)	0(1)
Δ268F-5a,d,m	76,732,559–76,791,779	66,964–126,184		0(3)	0(3)
Δ268F-6b,c,d	76,732,559–76,791,780	66,964–126,184		0(3)	0(3)
loxP-RT	76,858,743–76,858,758	N/A	N/A	N/A	N/A
Δ268-15a,c	76,860,843		2,085	0(2)	0(2)
Δ268-18a,d,m,n	76,982,805		124,047	0(4)	3(4)
Δ268-4c,f,k,q,s,t	76,994,171		135,413	3(3)	3*(6)
Δ268-4a,e,g,m,p,s	77,020,070		161,312	2(4)	2*(6)
Δ268-18t,x	77,113,970		255,212	1(2)	2(2)
Δ268-5c,d	82,480,936		5,622,178	2(2)	2*(2)
Δ268-4d	89,692,218		12,833,460	1(1)	0(1)

Chromosome 15 inversions		Inversion Size (base pairs)		DRT/DMC#	Instability^q^
Clone Name	Integration Site	Proximal	Distal	Clones +	Clones +
Inv268-3c	74,775,308	2,083,435		1(1)	1(1)
loxP-RT	76,858,743–76,858,758	N/A	N/A	N/A	N/A
Inv268-3a	77,502,462		643,704	0	1*(1)
Inv268-6c	77,643,709		784,951	1(1)	1(1)

# The number of clones that showed DRT/DMC (total clones scored).

q The number of clones that showed>10% of rearrangements of chromosome 15 (total clones scored).

*At least one clone with>90% of cells containing chromosome 15 rearrangements.

To characterize the Cre/loxP deletions in molecular detail, we first cloned and sequenced the original AP-loxP and loxP-RT cassette integration sites in P268 genomic DNA. The AP-loxP cassette is at position 54,407,235 base pairs of chromosome 16, and the loxP-RT cassette is at position 76,858,743 base pairs of chromosome 15 (NCBI Build 37/hg19). Subsequent analysis indicated that deletions in chromosome 15 displayed DRT/DMC (see below), while deletions in chromosome 16 did not. Therefore, we have concentrated on the chromosome 15 loxP-RT integration site in this report. In addition, because our random loxP integration approach generates deletions in only one direction (e.g. distal on chromosome 15; see Fig. 1E), we also modified the original loxP-RT integration site so that deletions could be generated proximal to the original loxP cassette (see [19] for details). Finally, we also characterized three different inversions in chromosome 15, which were generated by Lentiviral integrations of the loxP cassettes in the opposite orientation with respect to the original loxP-RT cassette integration site (see [21]).

**Figure 2:**
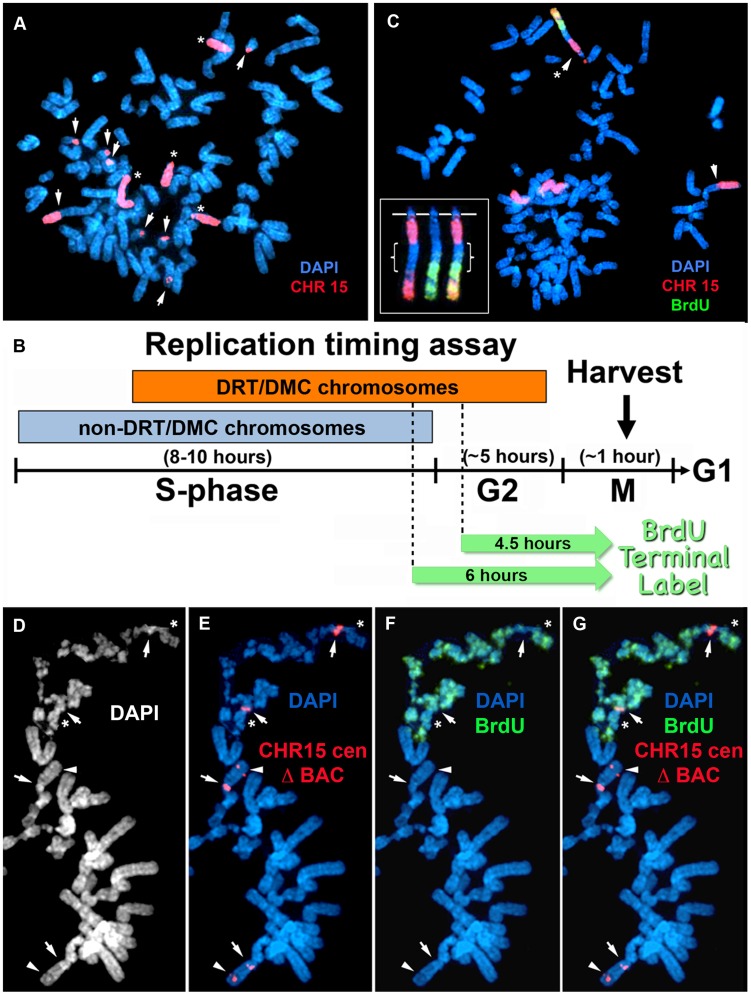
Chromosome rearrangements and delayed replication of a Cre/loxP-mediated deletion (∼135 kb) in chromosome 15. A) Secondary rearrangements of chromosome 15. Δ268-4f cells were processed for DNA FISH using a chromosome 15 WCP, and the DNA was stained with DAPI. Rearrangements involving chromosome 15 are indicated with arrows. Non-rearranged chromosome 15 s are indicated with asterisks. B) Schematic diagram of the BrdU Terminal Label replication-timing assay [23]. Cells were exposed to BrdU for either 4.5 or 6 hours, harvested for mitotic cells, and processed for BrdU incorporation and DNA FISH to identify chromosome 15. C) BrdU-WCP assay on cells containing an ∼135 kb distal deletion in chromosome 15. Δ268-4f cells were exposed to BrdU for 4.5 hours, harvested for mitotic cells, stained with an anti-BrdU antibody (green), and processed for DNA FISH with a chromosome 15 WCP (CHR 15; red). The DNA was stained with DAPI. Two different chromosome 15 secondary rearrangements are indicated with arrows. The inset shows the derivative chromosome 15 with the asterisk, with the BrdU staining and WCP shown in separate images. The brackets highlight the non-chromosome 15 DNA. D–G) BrdU-BAC assay on cells containing an ∼135 kb distal deletion in chromosome 15. Δ268-4c cells were exposed to BrdU for 4.5 hours, harvested for mitotic cells, stained with an anti-BrdU antibody (green), and processed for DNA FISH with a chromosome 15 centromeric probe (red) plus a BAC (CTD-2299E17; red) from the deleted region. The DNA was stained with DAPI (white in panel D or blue in panels E–G). The arrows mark the centromeric signals, and the arrowheads mark the BAC signals. The asterisks mark short arms of the deleted chromosome 15 s, which contain BrdU incorporation.

**Figure 3:**
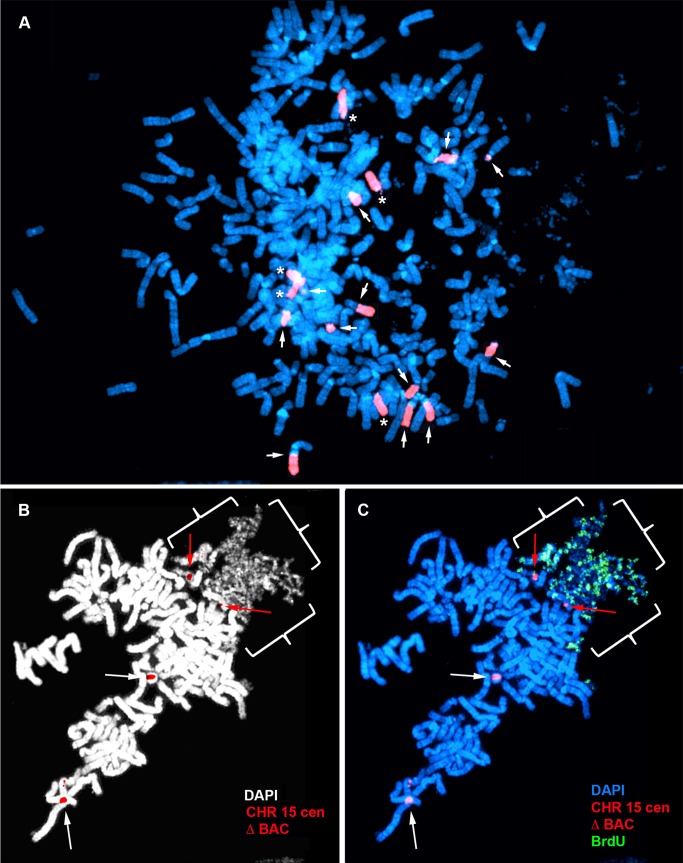
Chromosome rearrangements and delayed replication of a Cre/loxP-mediated deletion (∼5.6 mb) in chromosome 15. A) Secondary rearrangements of chromosome 15. Mitotic Δ268-5d cells were processed for DNA FISH using a chromosome 15 WCP, and the chromosomal DNA was stained with DAPI. Rearrangements involving chromosome 15 are indicated with arrows. Non-rearranged chromosome 15 s are indicated with asterisks. B and C) BrdU-BAC replication assay on cells containing the ∼5.6 mb distal deletion in chromosome 15. Δ268-5c cells were exposed to BrdU for 4.5 hours, harvested for mitotic cells, stained with an antibody to BrdU (green), and processed for DNA FISH with a chromosome 15 centromeric probe (red) plus a BAC (CTD-2299E17; ΔBAC, red) from the deleted region. The DNA was stained with DAPI (white in panel B or blue in panel C). The red arrows mark the centromeric signals from two chromosomes with DRT/DMC. The white arrows mark the centromeric signals from two non-DRT/DMC chromosome 15 s. The brackets highlight the extreme “pulverization” of two deleted chromosome 15 s.

**Figure 4:**
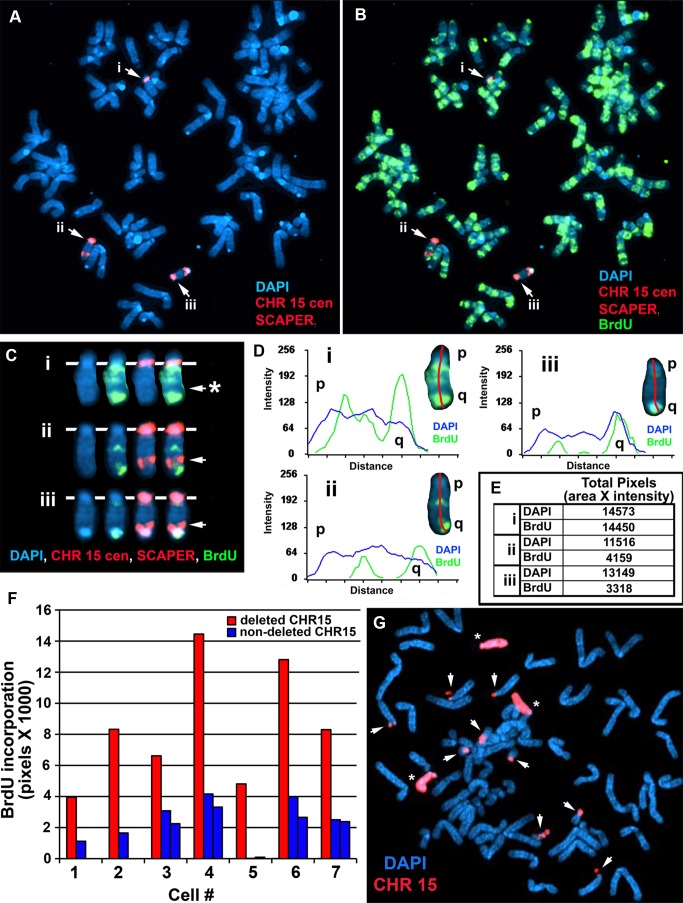
Delayed replication of chromosome 15 with an Cre/loxP-mediated ∼161 kb distal deletion. A–F) Δ268-4 g cells were incubated with BrdU for 6 hours, harvested for mitotic cells, stained with an antibody to BrdU (green) and processed for DNA FISH using a chromosome 15 centromeric probe (red) plus BAC CTD-2299E17 (red). The chromosomal DNA was stained with DAPI (blue). A and B) A metaphase spread containing three chromosome 15 s (i, ii, and iii). C) The three chromosome 15 s from panel B were cut out and aligned showing the BrdU and FISH signals in separate images. The asterisk marks the location of the deletion in the chromosome marked i, and the arrows mark the location of the BAC hybridization signals on chromosomes ii and iii. D) Pixel intensity profiles of the BrdU incorporation (green), and DAPI (blue) staining along the three chromosome 15 s from panel B. E) The pixel intensity (average intensity x area) for each chromosome, i, ii, and iii, showing the total amount of BrdU incorporation or DAPI staining. F) Quantification of the BrdU incorporation in multiple cells. The red and blue bars represent deleted and non-deleted chromosome 15 s, respectively, in 7 different cells. G) Instability of chromosome 15 containing an ∼161 kb Cre-loxP deletion. Mitotic Δ268-4e cells were processed for DNA FISH with a chromosome 15 WCP, and the chromosomal DNA was stained with DAPI. Rearrangements involving chromosome 15 are indicated with arrows, and non-rearranged chromosome 15 s are indicated with asterisks.

We characterized the Cre-dependent rearrangements in chromosome 15 at the genetic level using multiple independent assays, including: Southern blot hybridizations (S1 Fig.); PCR designed to span the genome-loxP-RT cassette junctions (S2A Fig. and B); LAM-PCR [22] to identify the Lentiviral 5′-LTR integration sites (Table 1; see [19]); PCR designed to span the Lentiviral-genome junctions (S2C Fig.); SNP analysis for loss of heterozygosity (S2D Fig.); and DNA FISH using BACs or fosmids located within the deleted or inverted regions (see Figs. 1E, 2D–G, 3B–C, 4A–C, S3A and C, S6A, and S8B and D Figs.). The largest deletion distal to the original loxP-RT cassette was ∼12.8 megabases (mb), and the smallest distal deletion was only ∼2 kilobases (kb) (see Table 1). The deletions proximal to the loxP-RT integration site did not cause DRT/DMC, and therefore were characterized genetically for loss of heterozygosity and with DNA FISH with a BAC from the deleted region (CTD-2117F7; see Fig. 1E), which allowed us to only determine the approximate sizes of the proximal deletions. Thus, the largest proximal deletion was ∼18 mb, and we characterized two different relatively small, ∼67–126 kb, proximal deletions. The genomic locations of all of the deletions and inversions characterized in this study are shown in Table 1. A schematic illustration of the loxP integration sites that generated five different distal deletions and two different inversions, all inducing DRT/DMC on chromosome 15, is shown in Fig. 1E.

### Structural Instability of chromosome 15

One complication in the analysis of chromosomes with DRT/DMC is structural instability of the affected chromosomes [7], [17], [18]. We previously used a “Luria-Delbruck fluctuation analysis” to characterize the instability associated with engineered chromosomes with DRT/DMC, and found that the t (15;16) generated in the P268 cell line results in an ∼80 fold increase in the rate of structural rearrangement of the affected chromosome [17]. In addition, once the DRT/DMC chromosomes become extensively rearranged they no longer display DRT/DMC [7], [16], [17]. Not surprisingly, the cells with DRT/DMC on chromosome 15, described in this report, acquired extensively rearranged chromosome 15 s and eventually lost the DRT/DMC phenotype during continued expansion in culture. Another complication in the analysis of DRT/DMC chromosomes is that they often experience non-disjunction events, resulting in cells that have either lost or gained the DRT/DMC chromosomes [16]. Furthermore, cells containing DRT/DMC chromosomes also experience endoreduplication at an increased frequency, resulting in an increase in the ploidy of the affected cells [16]. Consequently, during the characterization of the Cre/loxP-mediated rearrangements in chromosome 15, we observed additional copies of the Cre/loxP-rearranged chromosomes, and an increase in the ploidy of the cells with DRT/DMC chromosomes (see below).

To overcome the genome instability complications associated with DRT/DMC we employed a two-step process to characterize the stability and replication timing of the Cre/loxP-mediated rearrangements. First, we assayed low passage cultures for each clone, typically ≤5 passages, which limited the number of generations each clone was expanded through prior to the analysis. Second, we assayed each clone for rearrangements in chromosome 15 using DNA FISH with a whole-chromosome-paint (WCP) as probe, which allowed us to determine the frequency of secondary rearrangements in chromosome 15, and to identify clones with intact chromosome 15 s for detailed replication timing assays (see S1 Table).

An example of secondary rearrangements in chromosome 15 is shown in Fig. 2A. Mitotic spreads from Δ268-4f cells, which contain an ∼135 kb Cre/loxP-mediated distal deletion, were processed for DNA FISH using a chromosome 15 WCP. The chromosome 15 WCP detected 8 inter-chromosomal translocations involving chromosome 15 in this cell. In addition, the chromosome 15 WCP detected 4 non-rearranged chromosome 15s, indicating that this cell contained multiple copies of chromosome 15. Scoring additional metaphase cells with this WCP indicated that 25/50 (50%) cells in this clone contained structural rearrangements of chromosome 15, and the majority of the cells were also polyploid. Similar structural rearrangements affecting chromosome 15 were detected in clones containing Cre/loxP-mediated distal deletions≥124 kb (see below and S1 Table). In contrast, a similar DNA FISH analysis of two clones containing an ∼2 kb distal deletion did not reveal any new chromosome rearrangements involving chromosome 15, with 200 metaphase cells examined (S1 Table). Finally, an analysis of four independent sub-clones (generated by transfecting an *Aprt* expression vector, selection for Aprt+ cells, and clonal expansion) from parental P268 cells did not reveal any new rearrangements affecting chromosome 15 in any sub-clone, with 400 metaphase cells analyzed ([17]; and see S1 Table). This analysis indicates that transfection, selection for Aprt expression, and clonal expansion did not lead to structural instability of chromosome 15 in P268 cells.

### Deletions and inversions in chromosome 15 cause DRT/DMC

We next assayed replication timing of the chromosome 15 s in clones containing the various Cre/loxP-mediated rearrangements using a BrdU incorporation assay in combination with DNA FISH (see [23]). One critical component of this replication assay is the timing of the BrdU pulse. We used two different “terminal label” pulses of BrdU prior to mitotic harvest (see Fig. 2B). The shorter pulse, 4.5 hours, detected extremely late replication, essentially DNA synthesis during the G2 phase. This relatively short pulse of BrdU detects DNA synthesis on DRT/DMC chromosomes, but not on non-DRT/DMC chromosomes, as non-DRT/DMC chromosomes have finished replication prior to the pulse. The longer pulse, 6 hours, detects DNA synthesis during G2, but also detects the latest replicating DNA of non-DRT/DMC chromosomes at the end of S phase (see Fig. 2B). This longer pulse of BrdU allows us to compare differences in BrdU banding patterns between homologous chromosomes within the same cell, which allows for an estimate of the length of the delay, and allows for a quantitative assessment of replication timing differences between homologous chromosomes (see [23]).

We carried out two independent replication-timing assays on the clones characterized in this report. The first assay involved BrdU incorporation combined with DNA FISH with a chromosome 15 WCP as probe. This BrdU-WCP assay allowed us to assess replication timing of every chromosome 15 within a given cell, including any secondary rearrangements involving chromosome 15. The second assay combined BrdU incorporation with DNA FISH using a chromosome 15 centromeric probe plus BACs located within the Cre/loxP-deletions (see Fig. 1E). This BrdU-BAC assay allowed us to distinguish between the deleted and non-deleted chromosomes. However, the BrdU-BAC assay was not informative in cells with highly rearranged chromosome 15 s, as the secondary rearrangements did not always hybridize to the chromosome 15 probes.

An example of the BrdU-WCP assay in cells with an ∼135 kb distal deletion is shown in Fig. 2C. Cells from clone Δ268-4f were exposed to BrdU for 4.5 hours, harvested for mitotic cells, and processed for BrdU incorporation and DNA FISH with a chromosome 15 WCP. This mitotic cell was chosen to illustrate several points: 1) a single chromosome displays extremely late replication, the only detectable BrdU incorporation in this cell was in a derivative chromosome 15; 2) this cell contains 2 different chromosome 15 rearrangements, indicating that secondary rearrangements have already occurred on chromosome 15; and 3) the delayed replication on the chromosome 15 derivative also occurred on the translocated DNA from an unknown chromosome. These observations illustrate how an initial ∼135 kb deletion event can induce DRT/DMC, which subsequently causes structural instability of the affected chromosome, and that delayed replication can subsequently occur on the translocated DNA from a second chromosome.

An example of the BrdU-BAC assay on a second independent clone containing the ∼135 kb distal deletion is shown in Fig. 2D–G. For this analysis, Δ268-4c cells were exposed to BrdU for 4.5 hours, harvested for mitotic cells, processed for BrdU incorporation and subjected to DNA FISH using a chromosome 15 centromeric probe plus a BAC from the deleted region. The FISH signal from the centromeric probe allowed us to identify the chromosome 15 s, and the presence or absence of the BAC allowed us to distinguish between the non-deleted and deleted chromosomes, respectively. The only two chromosomes showing any detectable BrdU incorporation in this cell hybridized to the chromosome 15 centromeric probe but not to the BAC from the deleted region, indicating that the two chromosomes with DRT/DMC represent deleted 15 s. In addition, DNA synthesis was detected on both arms of the deleted chromosomes (Fig. 2D–G), indicating that the affects of the ∼135 kb deletion extend across the centromere. Also note that two other non-deleted chromosome 15 s, which hybridized to both the centromeric and BAC probes, did not retain any detectable BrdU incorporation, indicating that the non-deleted 15 s had finished replication prior to the BrdU exposure.

Another example of structural instability and DRT/DMC on chromosome 15 is shown in Fig. 3. Mitotic spreads from Δ268-5d cells, which contain one of our larger distal deletions (∼5.6 mb), were processed for DNA FISH using a chromosome 15 WCP. This probe detected 12 rearrangements involving chromosome 15 in this cell (Fig. 3A). Scoring multiple cells with the WCP indicated that>95% of the cells in this clone contained numerous secondary rearrangements of chromosome 15, and the majority of the cells were polyploid. Due to the high frequency of secondary rearrangements the replication timing assays were not performed in this clone. However, we did detect DRT/DMC in a second independent clone containing the same ∼5.6 mb deletion. Δ268-5c cells were exposed to BrdU for 4.5 hours, harvested for mitotic cells, processed for BrdU incorporation, and analyzed by DNA FISH using a chromosome 15 centromeric probe plus a BAC from the deleted region (Fig. 3B and C). Note the extreme level of ‘pulverization’ and ‘G2 DNA synthesis’ on two chromosomes that hybridized to the chromosome 15 centromeric probe. Also note the complete lack of BrdU incorporation in every other chromosome within this cell.

The delayed replication timing and pulverized appearance of the Cre/loxP-mediated deleted chromosomes shown above represent extreme examples of the DRT/DMC phenotype. However, this extreme level of delayed replication and condensation was detected in ≤5% of the mitotic cells in any given clone. Therefore, to determine if the cells that don′t display an extreme phenotype, yet retain intact chromosome 15 s, also display delayed replication we used the longer pulse of BrdU during the replication-timing assay (see Fig. 2B). The 6-hour pulse of BrdU allowed us to detect late DNA synthesis on DRT/DMC and non-DRT/DMC chromosomes and to directly compare the replication timing of the deleted and non-deleted chromosomes within the same cells. An example of this analysis is shown for clone Δ268-4 g, which contains an ∼161 kb distal deletion. Fig. 4A-E shows this analysis for a cell with three copies of chromosome 15, one with the Cre/loxP-mediated deletion and 2 without. Note that the deleted 15 contains a more extensive BrdU banding pattern than either non-deleted 15 (Fig. 4C). Comparing the BrdU banding pattern of the deleted 15 s to the pattern of non-deleted 15 s in multiple cells indicated that the replication timing of the deleted 15 s is delayed by≥2 hours. In addition, quantification of the BrdU incorporation indicated that the deleted 15 s were delayed in their replication timing in all seven cells analyzed (Fig. 4F). Furthermore, DNA FISH analysis with a chromosome 15 WCP as probe on a second clone containing the same ∼161 kb deletion, detected numerous secondary rearrangements of chromosome 15 (Fig. 4G), indicating that the ∼161 kb deletion also results in secondary rearrangements of chromosome 15. In contrast, cells containing the ∼124 kb distal deletion displayed a high degree of variability in BrdU incorporation in chromosome 15 s containing the ∼124 kb deletion. S3 Fig. shows this analysis for cells from clone Δ26818a, and indicates that certain cells in the population displayed a high degree of replication asynchrony between the deleted and non-deleted 15 s (S3A, B, and E Fig.), while other cells showed very little if any asynchrony (S3C, D, and E Fig.). Therefore, the ∼124 kb deletion results in variable expression of the DRT phenotype.

In contrast, similar DNA FISH and replication timing assays on parental P268 cells and in cells containing an ∼2 kb distal deletion indicated that the chromosome 15 s were structurally stable and replicated synchronously (Figs. S4–S5; and see S1 Table). Furthermore, the Cre/loxP-mediated deletions proximal to the original loxP-RT integration site, including two different deletions of ∼66–127 kb and an ∼18 mb deletion (see Table 1), did not cause delayed replication (S6 Fig.).

**Figure 5:**
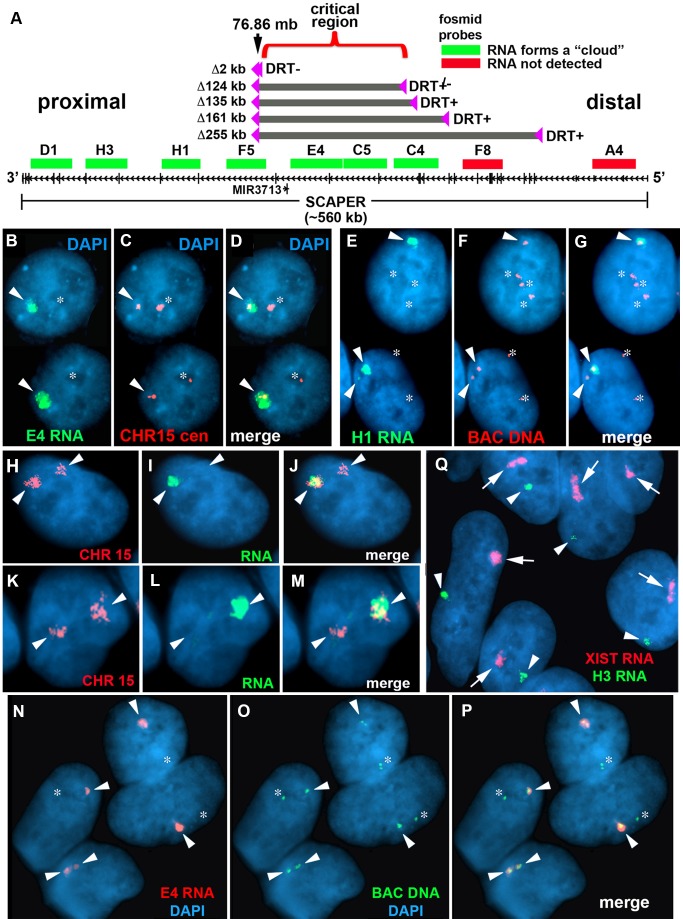
Differential allelic expression of *ASAR15*. A) A schematic diagram of *SCAPER*, *MIR3713*, RNA FISH probes, and five of the distal deletions in chromosome 15. The genomic location (in megabases), the exon-intron structure of *SCAPER*, the location of 9 fosmids [G248P87971D1 (D1), G248P81306H3 (H3), G248P82406H1 (H1), G248P87518F5 (F5), G248P82172E4 (E4), G248P8912C5 (C5), G248P89264C4 (C4), G248P88942F8 (F8), G248P80481A4 (A4)] used for RNA FISH, and the location of the five smallest distal deletions are shown. The fosmids that detect (green) or do not detect (red) RNA are indicated. B–D) Monoallelic expression of *ASAR15* in HTD114 cells. HTD114 cells were subjected to RNA FISH using a fosmid (E4 RNA; green) to detect RNA. Images of the RNA hybridization signals were obtained, and the coordinates of individual cells were recorded. Slides were subsequently processed for DNA FISH using a chromosome 15 centromeric probe (CHR15 cen; red) and new images of the DNA hybridization were captured for the same cells. E–G) P268 cells were processed for RNA-DNA FISH using the H1 probe to detect RNA, plus BAC CTD-2299E17 to detect DNA. H-M) RNA-DNA FISH using a pool of fosmid probes (D1, H3, H1, F5, E4, C5, and C4) to detect RNA (green) and a chromosome 15 paint to detect DNA (red). Panels H–J and K-M represent two different cells with the images shown in separate or merged panels. The arrowheads mark the chromosome 15s. N–P) Primary HFFs were processed for RNA-DNA FISH using probe E4 to detect RNA, plus BAC CTD-2117F7 to detect DNA. B–J) The nuclear DNA was stained with DAPI. Arrowheads mark the location of RNA signals, and asterisks mark the location of the DNA signals that lack corresponding RNA signals. In regions of the slides where the FISH worked well, the D1, H3, H1, F5, E4, C5, and C4 probes detected a positive signal in>90% of the HTD114, P268 and HFF cells. Q) RNA FISH in female HDFs. Female HDF cells were processed for RNA FISH using the H1 *ASAR15* probe (green) in combination with an XIST (red) probe. The arrows mark the large clouds of RNA detected by the *XIST* probe and the arrowheads mark hybridization signals detected by the *ASAR15* probe. The DNA was stained with DAPI.

While there is some variability in the replication timing between deleted and non-deleted chromosome 15 s in cells with deletions≥124 kb distal to the original loxP integration site, we did detect delayed replication timing and chromosome structure instability in clones from all of the distal deletions≥124 kb (Table 1 and S1 Table). In contrast, we did not detect delayed replication in P268 clones containg proximal deletions, an Aprt expression vector, nor in cells with the ∼2 kb distal deletion (see S1 Table). Therefore, the distal deletions define a relatively small critical region, ∼122 kb (see Fig. 5A), that when deleted results in delayed replication timing and instability of chromosome 15.

Next, to determine if chromosomal inversions at this same locus can also result in DRT/DMC we isolated P268 cells containing three different inversions generated at the original loxP-RT integration site in chromosome 15. For this analysis we used the random Lentiviral integration approach to isolate Aprt+ cells containing inversions (Inv268 clones) in both directions in chromosome 15 (see in Table 1). S7 Fig. shows BrdU-WCP assays on two different inversions in chromosome 15, and indicates that chromosomes containing inversions in either direction from the original loxP-RT cassette result in delayed replication and structural instability of chromosome 15. This result indicates that intra-chromosomal rearrangements without loss of DNA, in addition to deletions or translocations, can cause DRT/DMC and instability of chromosome 15.

### Differential expression of long RNA transcripts

The observations described above indicate that Cre/loxP-mediated rearrangements at a discrete *cis*-acting locus cause DRT/DMC and structural instability of human chromosome 15. Because the phenotype of the Cre/loxP-rearranged chromosome 15 s is similar to the phenotype induced by disruption of either *Xist* or *ASAR6* [19], [24] and *Xist* and *ASAR6* encode large monoallelically expressed non-coding RNAs, we next assayed expression of the chromosome 15 locus using RNA-DNA FISH. Because the smallest deletion that results in DRT/DMC on chromosome 15 is located entirely within the large protein-coding gene *SCAPER* (see Fig. 5A), we used multiple probes from within the *SCAPER* coding region (spanning ∼560 kb of genomic DNA) to detect RNA, plus either a chromosome 15 centromeric probe, BACs from nearby, or a chromosome 15 paint to detect the DNA. Fig. 5B–D shows an example of this analysis for parental HTD114 cells using a probe from within the deleted region (E4) to detect RNA, plus a chromosome 15 centromeric probe to detect the DNA. Note the large “cloud” of RNA detected on one copy of chromosome 15. An example of RNA-DNA FISH in P268 cells using the H1 probe to detect RNA is shown in Fig. 5E–G. Note that these two cells are tetraploid for chromosome 15 and that the H1 probe detects RNA expressed from only one or two of the chromosome 15 s. We also note that the size of the RNA hybridization signals detected by these probes was variable, ranging form large “clouds” to relatively small pinpoint sites of hybridization (see Fig. 5B–G). In addition, we detected similar RNA hybridization signals using the D1, H3, F5, C5, and C4 probes, and hybridization was detected on ∼50% of the chromosome 15 s in P268 cells (see Fig. 5A). Therefore, the D1, H3, H1, F5, E4, C5 and C4 probes, spanning ∼370 kb of genomic DNA, detect transcripts that are retained within the nucleus and are localized to one of the chromosome 15 homologs. In addition, to determine if the large clouds of RNA detected from within *SCAPER* are physically localilzed to chromosome 15 we carried out RNA-DNA FISH using a pool of fosmids (D1, H3, H1, F5, E4, C5 and C4) to detect RNA plus a chromosome 15 paint to detect DNA. Fig. 5H–M shows a panel of cells with large RNA signals that is localized to single chromosome 15 territories within the nuclei. We detected chromosome-sized RNA FISH signals in 82% (89/108) of P268 cells using this pool of fosmid probes.

In contrast to the D1, H3, H1, F5, E4, C5 and C4 probes, we were not able to detect RNA using the F8 or A4 probes in either HTD114 or P268 cells (Fig. 5A and see S8 Fig.). Thus, even though the F8 and A4 probes are within *SCAPER,* and are capable of detecting DNA as efficiently as the E4 probe using DNA FISH on metaphase chromosomes (see S8 Fig.), they were not able to detect expression of RNA in HTD114 or P268 cells. However, we did detect low-level expression of the 5′ region of properly spliced *SCAPER* mRNA using RT-PCR (see S11 Fig.). Therefore, the 5′ region of *SCAPER* distal to the C4 probe (∼150 kb) is expressed, but not at high enough levels to be detected by RNA FISH using the F8 or A4 probes.

Next, to determine if similar clouds of transcripts are present in human primary cells we used RNA-DNA FISH on human foreskin fibroblasts (HFFs). Fig. 5N–P shows an example of this analysis using the E4 probe to detect RNA, plus a BAC to detect the DNA from this locus. The E4 probe detected a single large RNA signal on one chromosome 15 in ∼76% (41/54) of cells, or on both copies of chromosome 15 in ∼24% (13/54) of cells (Fig. 5H–J). Similar results were obtained with the D1, H3, H1, F5, C5 and C4 probes (see Fig. 5A. In contrast, but similar to HTD114 and P268 cells, the F8 and A4 probes failed to detect RNA in HFFs (see Fig. 5A). In addition, to directly compare the size and appearance of the RNA signal detected from within *SCAPER* to XIST RNA on the inactive X chromosome we analyzed expression of the chromosome 15 locus plus XIST simultaneously in female primary dermal fibroblasts HDFs. Figs. 5Q and S9E and S9F show RNA detected by the H3 probe in relation to RNA detected by the *XIST* probe. We detected chromosome-sized RNA FISH signals in 72% (72/100) of primary HDF cells.

In addition, we also detected expression of RNA from ∼50% of the chromosome 15 s in human HepG2 cells (S9A–D Fig.). Furthermore, analysis of RNA-seq data, generated by the Encode Project, indicated that RNA expressed from the critical region for DRT/DMC, defined above, is enriched in the nuclear Poly A minus fraction in HepG2 cells (S10 Fig.). We previously found that ASAR6 RNA is present in the Poly A minus fraction and does not contain introns yet is transcribed by RNA Polymerase II [18]. Therefore, to determine if the RNA produced from within the critical region of *SCAPER* is the product of RNA Polymerase II, we analyzed expression of RNA in cells treated with α-amanitin, which is a selective inhibitor of RNA Polymerase II [25], using a semi-quantitative RT-PCR assay. S11 Fig. shows the results of this analysis, and indicates that the RNA derived from the critical region is indeed sensitive to α-amanitin. Similarly, RNA expressed from the protein-coding gene *P300* is also sensitive to α-amanitin treatment. We also detected properly spliced SCAPER mRNA and found that it was also sensitive to α-amanitin treatment. In contrast, expression of 45S RNA (an RNA Polymerase I product) and a tRNA gene (an RNA Polymerase III product) were not inhibited by α-amanitin. We conclude that the RNA produced from the critical region for DRT/DMC is generated by RNA Polymerase II. In addition, this analysis indicated that the half-life of the RNA from within SCAPER introns 21, 23, and 24 is similar to the half-life of ASAR6 RNA, which is approximately 5 hours. In addition, the half-life of these intronic RNAs is much longer than the half-life of properly spliced P300 or SCAPER mRNAs (∼2 hours), which are spliced, polyadenylated, RNA Polymerase II products.

Taken together, our observations indicate that RNA transcripts from an ∼370 kb region from within the *SCAPER* gene are retained within the nucleus, and can be detected as chromosome sized clouds of RNA localized to either one homolog of human chromosome 15 in human cell lines and in primary skin fibroblasts. However, whether these transcripts represent transcription initiating within *SCAPER*, or represent transcripts that are processed from the larger *SCAPER* primary transcript and preferentially retained on chromosome 15 is currently not known. Regardless, because the locus that encodes these transcripts also displays asynchronous replication (see below), we have named these transcripts ASynchronous replication and Autosomal RNA on chromosome 15, or ASAR15.

### Disruption of the expressed allele of *ASAR15* results in DRT/DMC

**Figure 6:**
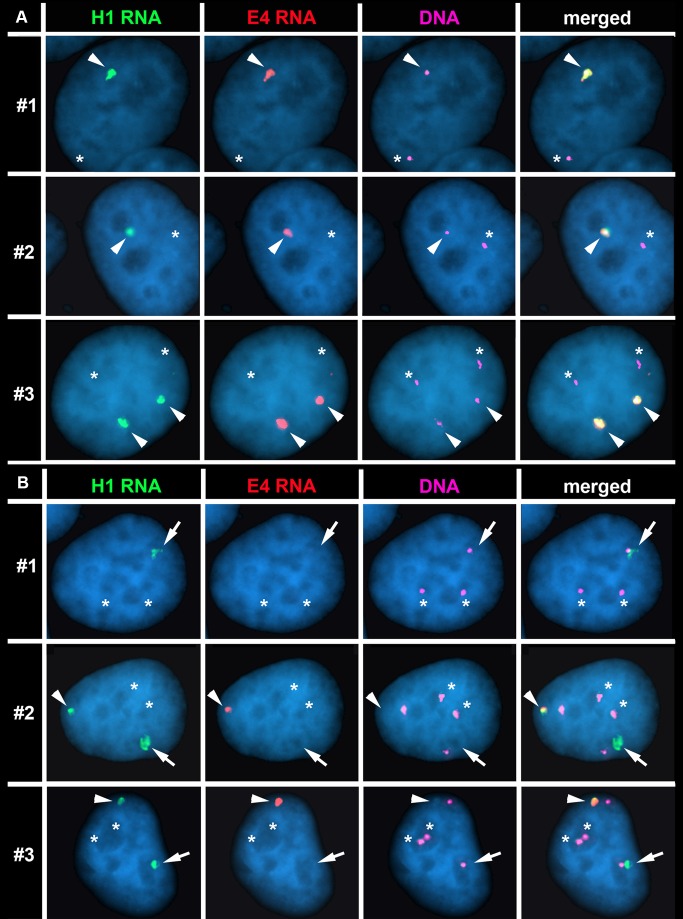
The Cre/loxP-mediated deletions occurred on the expressed allele of *ASAR15*. P268 (panel A) and Δ268-4 g (panel B) cells were subjected to RNA FISH using the H1 (green) and E4 (red) probes to detect RNA. Images of the RNA hybridization signals were obtained, and the coordinates of individual cells were recorded; the slides were subsequently processed for DNA FISH (BACs CTD-2299E17 plus BAC-CTD-2117F7) and new images of the DNA hybridization were captured for the same cells. The DNA FISH step included an RNAase step, which eliminated the RNA FISH signals. The DNA FISH hybridization signal was pseudo-colored purple for clarity, and the nuclear DNA was stained with DAPI (blue). Representative images from three different P268 and Δ268-4 g cells (#1–3) are shown in each panel (A and B). The arrowheads mark the coincident sites of hybridization detected by both probes. The arrows mark the sites of hybridization with the H1 probe that was not coincident with a site of hybridization with the E4 probe in Δ268-4 g cells (panel B). The asterisks mark the sites of DNA hybridization that lacked corresponding RNA hybridization signals from either H1 or E4 probes.

Previous studies indicate that disruption of the expressed alleles of either *Xist* or *ASAR6* result in extremely late replication and structural instability of their respective chromosomes [19], [24]. Therefore, to determine if the deletions that cause DRT/DMC and instability on chromosome 15 occurred on the chromosome retaining ASAR15 RNA we utilized an RNA-DNA FISH assay that allowed us to distinguish between the deleted and non-deleted chromosomes. For this analysis we used a probe from within the deletion (E4) plus a probe from outside the deletion (H1) simultaneously to detect RNA in cells containing the ∼161 kb distal deletion (see Fig. 5A). Thus, if the deletion occurred on the silent copy of chromosome 15, the H1 and E4 probes would detect ASAR15 RNA concomitantly. In contrast, if the deletion occurred on the expressed allele, the E4 probe would not detect RNA from the deleted 15, but the H1 probe would still detect RNA expressed from the deleted chromosome. First, to determine if the H1 and E4 probes detect the same sites of expression in non-deleted cells, we assayed expression of RNA in P268 cells using both probes simultaneously. Fig. 6A shows examples of this analysis and indicates that both probes detected RNA from the same chromosome 15. Note that the P268 cells in Fig. 6A are either diploid (cell #1 and #2) or tetraploid (cell #3) for chromosome 15, and hybridization with both probes was detected on either one or two chromosome 15 s, respectively. We detected simultaneous expression with the H1 and E4 probes in 100% (50/50) of P268 cells. Therefore, the H1 and E4 probes detect expression from the same chromosome 15 s.

Next, to determine if the rearrangements that cause DRT/DMC affect the expressed or non-expressed allele of *ASAR15* we processed Δ268-4 g cells for RNA-DNA FISH using the H1 and E4 probes to detect RNA. Fig. 6B shows examples of this analysis and indicates that the H1 probe detected clouds of RNA, which are indistinguishable in number and appearance to the clouds expressed in parental P268 cells (Fig. 6A). Note that the Δ268-4 g cells in Fig. 6B are either triploid (cell #1) or tetraploid (cell #2 and #3) for chromosome 15, and the H1 probe detected RNA from one (cell #1) or two (cell #2 and #3) chromosome 15 s, respectively. In contrast, the E4 probe detected zero (cell #1) or one (cell #2 and #3) site of expression in these same cells, indicating that the RNA detected by the E4 probe was absent from one of the alleles detected by the H1 probe in 100% (50/50) of the cells. Therefore, the Cre/loxP-mediated deletion occurred on the expressed allele of *ASAR15*. In addition, because the H1 probe detected expression from the deleted chromosome, the ∼161 kb deletion did not disrupt expression nor retention of transcripts proximal to the original loxP-RT integration site (see Fig. 5A). Finally, because the E4 probe detected RNA in some of the cells in Δ268-4 g (cell #2 and #3), some of the Δ268-4 g cells retain an intact copy plus a deleted copy of the expressed allele of *ASAR15*, which was not surprising as the cells that still express RNA detected by the E4 probe were tetraploid for chromosome 15 (Fig. 6B). Regardless, these observations indicate that *ASAR15* RNA is present on one chromosome 15 homolog in P268 cells, and that the Cre/loxP rearrangements that cause DRT/DMC occurred on the chromosome 15 that retained ASAR15 RNA.

### Asynchronous replication

Because all monoallelic genes display asynchronous replication between alleles (reviewed in [26]), we next tested whether the locus that encodes ASAR15 RNA also displays asynchronous replication. For this analysis we used a DNA FISH based assay [27] to determine the extent of asynchronous replication on chromosome 15. This assay uses probes to particular chromosomal locations hybridized to cells in S-phase. This assay also includes a methanol/acetic acid fixation step, which destroys the nuclear architecture and allows for a relatively accurate assessment of replication synchrony [28], [29]. The hybridization signals are present in nuclei in three distinct patterns, which are dependent on the replication status of each allele. The first pattern corresponds to two single hybridization dots (the SS pattern), indicating that neither allele has replicated. The second pattern corresponds to two double dots (the DD pattern), indicating that both alleles have replicated. The third pattern corresponds to one single dot and one double dot (the SD pattern), indicating that only one of the alleles had replicated. For asynchronously replicating loci the SD pattern is present in 30–50% of nuclei. In contrast, synchronously replicating loci show the SD pattern in only 10–20% of nuclei [29]–[31]. We used this “single dot-double dot” assay in primary HFFs to determine the degree of replication asynchrony on chromosome 15. We found that two different non-overlapping probes (BACs CTD-2299E17 and CTD-2117F7; see Fig. 1E) from within the *ASAR15* region, as well as three additional chromosome 15 random monoallelic genes (*MYO1E, PTPN9,* and *PEAK1;* [32], [33]) located ∼1-20 mb from *ASAR15*, all display a high percentage of the SD pattern, which is indicative of asynchronous replication (Table 2).

**Table 2:** DNA FISH analysis of human loci.

				Coordination	
Locus	Probe	Position	%SD	cis	trans
*MYO1E*	RP11-1089J12	15q22.2	40	72%	28%
*PTPN9*	CTD-2323K18	15q24.2	48	71%	29%
*ASAR15*	CTD-2117F7	15q24.3	35	89%	11%
*ASAR15*	CTD-2299E17	15q24.3	47	XX	XX
*PEAK1*	RP11-94P14	15q24.3	35	74%	26%
*PTK6*	RP11-95N13	20q13.3	19	nd	nd
*LARP*	CTD-2546N4	5q33.2	24	nd	nd
*C9orf43*	CTD-2167I10	9q32	19	nd	nd

The percentage of the single-double (%SD) pattern was determined using DNA FISH.

Coordinated asynchronous replication was scored against BAC CTD-2299E17.

Previous studies found that the asynchronous replication of random monoallelic genes on any given chromosome displays coordination either in *cis* or in *trans* [18], [19], [30], [31], [34]. Therefore, we next determined if the asynchronous replication of *ASAR15* is coordinated with other monoallelic loci on chromosome 15. For this analysis we used a two-color DNA FISH assay to determine the SD pattern for two loci simultaneously. We used a probe representing *ASAR15* in combination with BAC probes for *MYO1E, PTPN9*, and *PEAK1* (see Table 2). This analysis indicated that the asynchronous replication of *ASAR15* was coordinated in *cis* with *MYO1E* (72/100 cells, *P*<1×10^−4^), *PTPN9* (71/100 cells *P*<1×10^−4^), and *PEAK1* (74/100 cells, *P*<1×10^−5^).

**Figure 7:**
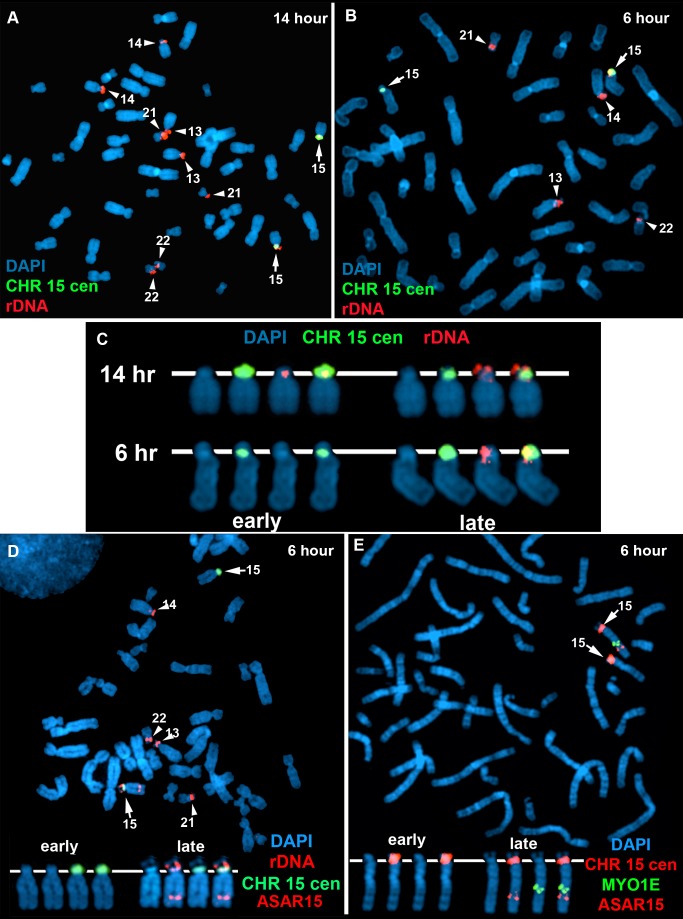
Coordinated random asynchronous replication on chromosome 15. A–C) ReTiSH assay on rDNA loci in PBLs. PBLs were labeled with BrdU for 14 (A) or 6 (B) hours, arrested in metaphase, and subjected to ReTiSH using an 18S rDNA probe (red). The chromosome 15 s were identified using a centromeric probe (green), and the chromosomal DNA was detected with DAPI. A and B) The DAPI images of the chromosomes were inverted and the banding patterns were used to identify all of the ReTiSH positive chromosomes. The arrows mark the chromosome 15 s, and the arrowheads mark the other four chromosomes containing rDNA clusters (13, 14, 21, and 22). C) The ReTiSH signals for the rDNA (red) and chromosome 15 centromeric (green) probes from the 14 (panel A) and 6 (panel B) hour time points are shown. The early and late replicating chromosome 15 s are indicated for the 6 hour time point. D) ReTiSH assay using an *ASAR15* BAC (CTD-2299E17; red), an rDNA probe (red), and a chromosome 15 centromeric probe (green). The *ASAR15* and rDNA probes show hybridization signals to the same chromosome 15 homolog at the 6 hour time point. E) ReTiSH assay using an *ASAR15* BAC (CTD-2299E17; red), a *MYO1E* BAC (RP11-1089J12; green) and a chromosome 15 centromeric probe (red). The *ASAR15* BAC and the *MYO1E* BACs show hybridization signals to the same chromosome 15 homolog at the 6 hour time point. D and E) The early and late replicating chromosome 15 s are indicated.

One limitation of the DNA FISH assay described above is that probes on the same chromosome that are>50 mb apart are difficult to score, as the hybridization signals coming from one homolog may be closer to hybridization signals coming form the other homolog. To overcome this limitation, we utilized a second replication-timing assay known as Replication Timing-Specific Hybridization, or ReTiSH [34]. The ReTiSH assay involves BrdU incorporation for different times followed by the analysis of metaphase chromosomes using a modification of the Chromosome Orientation-Fluorescence In Situ Hybridization (CO-FISH) assay [35]. CO-FISH involves the conversion of BrdU incorporated chromosomal DNA into single stranded DNA followed by hybridization with specific probes without further denaturation of the chromosomal DNA. Because metaphase spreads are analyzed for hybridization signals along individual chromosomes, the distance between the loci is not a limitation of the ReTiSH assay [34]. In order to assay asynchronous loci along the length of chromosome 15 we used probes for *ASAR15* (15q24), the rDNA cluster (15p11), and MYO1E (15q22). The rDNA clusters are located on five different human chromosomes (13, 14, 15, 21 and 22), and were recently shown to display random asynchronous replication [34]. Hybridization of the rDNA probe to chromosomes 13, 14, 15, 21, and 22 also served as an internal control for asynchronous replication on multiple chromosomes detected simultaneously by the ReTiSH assay. This assay also included a chromosome 15 centromeric probe, which allowed for the unambiguous identification of the chromosome 15 s. Centromeric heterochromatin is known to be late replicating, and the ReTiSH assay detects hybridization of centromeric probes to both homologs at both time points [34]. For this analysis we exposed human primary blood lymphocytes (PBLs) to BrdU for either 14 or 6 hours and then processed the cells for ReTiSH. Fig. 7A–C shows that the rDNA probe hybridized to both homologs of chromosomes 13, 14, 15, 21, and 22, at the 14 hour time point, but hybridization to single homologs of these same chromosomes at the 6 hour time point. Therefore, our ReTiSH assay detected asynchronous replication of the rDNA clusters on all five chromosomes. We detected hybridization of the rDNA probe to both copies of chromosome 15 in 100% (50/50) of cells at the 14 hour time point, and to a single copy of chromosome 15 in 100% (50/50) of cells at the 6 hour time point, which is consistent with previous observations that the rDNA cluster on chromosome 15 displays asynchronous replication [34]. Similarly, we found two sites of hybridization for *ASAR15* or *MYO1E* at the 14 hour time point (50/50 cells for both probes), and single sites of hybridization for *ASAR15* (47/50 cells), or *MYO1E* (47/50 cells) at the 6 hour time point, indicating asynchronous replication at both loci. Next, we used a two-color hybridization scheme to simultaneously detect *ASAR15* plus rDNA or *MYO1E*. We found that the asynchronous replication of *ASAR15* was coordinated in *cis* with the rDNA cluster on chromosome 15 (Fig. 7D; 44/50 cells, *P*<1×10^−5^), and with the *MYO1E* gene (Fig. 7E; 43/50 cells, *P*<1×10^−5^). Therefore, the asynchronous replication of *ASAR15* is coordinated in *cis* with other monoallelic loci located>70 mb away and on both sides of the centromere.

## Discussion

Mammalian cells replicate their chromosomes every cell cycle during a defined temporal replication program (reviewed in [36]). Recent studies indicate that at least half of the genome is subject to changes in the replication timing of relatively large chromosomal domains along every chromosome during normal development [37], [38]. However, the determinants that control replication timing are poorly understood, and are not encoded within the DNA sequence of the origins. The current thinking is that the timing of origin firing is dictated by chromosomal location, and is directly linked to complex higher-order features of chromosome architecture [39], [40]. In addition, recent studies have identified DNA binding proteins that can dictate origin timing and implicate the spatial organization of origins within nuclear territories in the mechanism of replication timing control (reviewed in [41], [42]).

In this report we found that chromosomal rearrangements at a discrete *cis*-acting locus on human chromosome 15 cause delayed replication of the entire chromosome. This locus represents the third example of a *cis*-acting locus that, when disrupted, results in delayed replication of an entire mammalian chromosome, with mouse *Xist* and human *ASAR6* representing the other two loci [19], [24]. The chromosome 15 locus described here is complex, and encodes the large protein-coding gene *SCAPER*, the micro RNA gene *MIR3713*, and large, preferentially retained, nuclear transcripts (ASAR15) that can be detected as chromosome-sized clouds on chromosome 15. Whether or not the ASAR15 transcripts are initiated within *SCAPER*, or represent transcripts that are processed from the larger *SCAPER* primary transcript and preferentially retained on chromosome 15 is currently not known. Regardless, the identification of loci, (*Xist*, *ASAR6*, and *ASAR15*) that control replication timing of three different chromosomes suggests that the replication-timing program of all mammalian chromosomes involves differentially expressed, asynchronously replicating, cis-acting, non-coding RNA genes.

Monoallelic gene expression in mammals occurs in two distinct patterns, random and imprinted (reviewed in [26]). The inequality of the two alleles in both patterns is characterized by differential DNA methylation, differential chromatin modifications, unequal nuclear localization, and expression of large non-coding RNAs [26], [43]–[45]. While parallels between X inactivation and the *cis*-coordinated replication asynchrony of autosomal random monoallelic genes have been made [26], [30], [31], [34], [46], [47], recent reports indicate that not all autosomal random monoallelic genes are expressed from the same homolog in some clonal cell lines, or that these same genes may be biallelically expressed in other clones [33], [48], [49]. Our RNA FISH analysis of ASAR15 RNA expression in HTD114, P268, and HepG2 is consistent with monoallelic expression of this locus in these clonal cell lines. However, a similar RNA FISh analysis of non-clonal primary fibroblasts revealed that at least some cells in the population contain biallelic expression of *ASAR15*. Therefore, *ASAR15* appears to display similar characteristics as other random autosomal “monoallelic” genes, in that at least some cells display biallelic expression.

Another characteristic of all monoallelic genes, both random and imprinted, is that they display asynchronous replication between alleles located on homologous chromosomes [26], [29]–[31], [50]. Asynchronous replication with random choice represents an epigenetic state that is established early in development, is present in all tissues, and is independent of gene expression [29]–[31]. In this report, we used two different replication-timing assays, ‘single dot-double dot′ and ReTiSH, to assess replication synchrony along human chromosome 15 in two different primary cell types, HFFs and PBLs. We found that the asynchronous replication of *ASAR15* is coordinated in *cis* with other random monoallelic loci separated by>70 mb of genomic DNA, and located on either side of the centromere. Furthermore, because the asynchronous replication of the rDNA locus on chromosome 15 is random and not imprinted [34] and the asynchronous replication of *ASAR15* is coordinated in *cis* with the rDNA locus, the asynchronous replication of *ASAR15* must also be random.

Another common feature of monoallelic genes is a relatively high density of LINE-1 elements [51]–[53]. Consistent with this observation, the *ASAR6* and *ASAR15* loci contain a high concentration of LINE-1 elements within the expressed regions of each locus, constituting>40% and>55% of the sequence, respectively. Note that in humans, autosomes contain on average ∼17.6% LINE-1 sequence and the X chromosome contains ∼31.0% LINE-1 sequence [53]. In addition, recent observations indicate that LINE1 RNA shares at least some characteristics with XIST RNA [54]. While LINE-1 elements have been implicated in monoallelic gene expression on the X chromosome and on autosomes for many years [51]–[53], [55]–[57], a mechanistic role for these abundant elements during transcriptional silencing and/or replication timing has not been established.

Previous studies indicate that *ASAR6* and *Xist* share many important physical and functional characteristics [18], [19]. In this report we found that *ASAR15* also shares many of these same characteristics, including: 1) differential expression of large RNA transcripts that are retained in the nucleus, 2) random asynchronous replication that is coordinated with other linked monoallelic genes, and 3) disruption of the expressed allele results in delayed replication timing and structural instability of an individual mammalian chromosome. In addition, ASAR15 RNA was detected as chromosome-sized clouds on one homolog of chromosome 15, and that these clouds are similar in appearance to XIST RNA localized to the inactive X chromosome.

The molecular mechanisms by which *Xist*, *ASAR6* or *ASAR15* control replication timing of entire chromosomes is currently not known. Interestingly, disruption of the transcriptionally silent mouse *Xist* gene, in male or female somatic cells, results in a small but significant delay in replication of the active X chromosome, indicating that Xist RNA expression is not involved in the delayed replication of the active X chromosome following *Xist* gene disruption [58]. In contrast, disruption of the expressed *Xist* allele, on the inactive X chromosome, results in extremely late replication, abnormal chromatin structure, and instability of the X chromosome [24]. Therefore, whether or not the *Xist* gene is transcribed has a dramatic affect on the severity of the replication timing delay and stability of the X chromosome, implicating the act of transcription or the Xist RNA in maintaining proper replication timing of the inactive X chromosome [24]. We have found that disruption of the expressed alleles of either *ASAR6* or *ASAR15* results in DRT/DMC and structural instability of their respective chromosomes ([18], [19]; and see above]. Whether or not the transcriptionally silent alleles of *ASAR6* or *ASAR15* control replication timing of their respective chromosomes is currently not known. Furthermore, ASAR15 RNA could be detected as a chromosome-sized cloud of RNA in human cell lines and in primary skin fibroblasts. In contrast, *ASAR6* RNA does not form large clouds on chromosome 6 in clonal cell lines nor in primary blood lymphocytes [18], [19]. Therefore, the significance of the presence or absence of large clouds of RNA expressed from *ASAR15* or *ASAR6* remains an open question.

One complication in the analysis of chromosomes with DRT/DMC is the dramatic genomic instability that occurs in cells with these abnormal chromosomes. There are at least two distinct types of genomic instabilities that have been described in cells with DRT/DMC chromosomes. First, and the best characterized, is chromosome structure instability (CSI). The CSI associated with DRT/DMC is characterized by a 30–80 fold increase in the rate of chromosome rearrangements affecting the DRT/DMC chromosomes [17]. Because the secondary rearrangements that occur on the DRT/DMC chromosomes are often inter-chromosomal translocations, all of the chromosomes within a cell with DRT/DMC chromosomes can be affected [17]. The second type of genomic instability associated with DRT/DMC is chromosome instability (CIN). There are two distinct manifestations of CIN in cells with DRT/DMC chromosomes. First, DRT/DMC chromosomes activate the spindle assembly checkpoint, are often observed as lagging chromosomes during anaphase, and experience non-disjunction events at an increased frequency resulting in gains and losses of the DRT/DMC chromosomes [16], [18]. Second, cells with DRT/DMC chromosomes also experience failed cytokinesis, which results in endoreduplication and dramatic whole chromosome copy number alterations [16]. Therefore, the CIN that occurs in cells with DRT/DMC chromosomes can also affect the copy number of non-DRT/DMC chromosomes within the same cell. Therefore, the CSI and CIN observed in cells with DRT/DMC chromosomes affects the stability of the entire genome [1].

We previously used IR to generate chromosome rearrangements in mouse and human cells, and found that ∼5% of inter-chromosomal translocations involving many different autosomes displayed DRT/DMC [15]. In addition, we detected DRT/DMC in primary cells derived from skin fibroblasts, blood lymphocytes, and kidney epithelial cells, indicating that the DRT/DMC phenotype can affect chromosomes present in non-transformed cells from three different tissues. Furthermore, our original chromosome-engineering screen for DRT/DMC chromosomes identified five balanced translocations, affecting eight different autosomes, all displaying DRT/DMC on at least one of the two derivative chromosomes [17]. We previously characterized one of these balanced translocations in molecular detail and found that disruption of the *ASAR6* gene results in DRT/DMC on chromosome 6 [19]. In this report, we characterized a second balanced translocation from this screen and identified a *cis*-acting locus that when disrupted results in DRT/DMC on a second human autosome. Our work, combined with the observation that disruption of *Xist* results in a similar replication and instability phenotype [24], suggests that all mammalian chromosomes contain *cis*-acting loci that function to synchronize chromosome-wide replication timing, promote monoallelic gene expression and help maintain structural stability of individual chromosomes [1], [18], [19]. We believe that these *cis*-acting loci are as fundamentally important to mammalian chromosome function as telomeres, centromeres, or origins of replication. Therefore, we propose that every mammalian chromosome contains four essential *cis*-acting elements: origins, telomeres, centromeres, and “inactivation/stability centers”, all functioning to ensure proper replication, segregation and stability of each chromosome.

## Materials and Methods

### Cell culture

The methods for culturing the cells in this study were carried out as previously described [18]. Low passage primary human (male) foreskin fibroblasts (HFFs) were obtained from ATCC and cultured in DMEM plus 10% fetal bovine serum (Hyclone). Primary, human (female) dermal skin fibroblasts (HDFs) were provided by Dr. Shoukhrat Mitalipov. Primary blood lymphocytes were isolated after venipuncture into a Vacutainer CPT (Becton Dickinson, Franklin Lakes, NJ) per the manufacturer's recommendations and grown in 5 mL RPMI 1640 (Life Technologies) supplemented with 10% fetal bovine serum (Hyclone) and 1% phytohemagglutinin (Life Technologies). HTD114 and P268 cells are *APRT* deficient cell lines derived from the HT1080 fibrosarcoma [59], and were grown in DMEM (Gibco) supplemented with 10% fetal bovine serum (Hyclone). P268 derivatives were grown as above with the addition of 500 mg/ml Geneticin (Gibco), 200 mg/ml Hygromycin B (Calbiochem), and/or 10 ug/ml Blasticidin S HCl (Invitrogen). The deletion-line derivatives were grown in DMEM supplemented with 10% dialyzed fetal bovine serum (Hyclone), 10 mg/ml azaserine (Sigma) and 10 mg/ml adenine (Sigma) to facilitate selection for *Aprt*-expressing cells. All cells were grown in a humidified incubator at 37°C in a 5% carbon dioxide atmosphere.

### DNA FISH

The methods for DNA FISH assays in this study were carried out as previously described [18]. Trypsinized cells were centrifuged at 1,000 rpm for 10 minutes in a swinging bucket rotor. The cell pellet was re-suspended in 75 mM potassium chloride for 15–30 minutes at 37°C, re-centrifuged at 1,000 rpm for 10 minutes and fixed in 3∶1 methanol∶acetic acid. Fixed cells were added drop-wise to microscope slides to generate mitotic chromosome spreads using standard methods [60]. Slides with mitotic spreads were baked at 85°C for 20 minutes and then treated with 0.1 mg/ml RNAase for 1 hour at 37°C. After RNAase treatment, the slides were washed in 2xSSC (1xSSC is 150 mM NaCl and 15 mM sodium citrate) with 3 changes for 3 minutes each and dehydrated in 70%, 90%, and 100% ethanol for 3 minutes each. The slides were denatured in 70% formamide in 2xSSC at 70°C for 3 min and whole chromosome paints were used according to the manufacturer's recommendations and hybridization solutions (American Laboratory Technologies and Vysis). Detection of digoxigenin-dUTP probes utilized a three-step incubation of slides with sheep FITC-conjugated anti-digoxigenin antibodies (Roche) followed by rabbit FITC-conjugated anti-sheep antibodies (Roche) followed by goat FITC-conjugated anti-rabbit antibodies (Jackson Laboratories). Slides were stained with DAPI (12.5 mg/ml) or propidium iodide (0.3 mg/ml), cover slipped, and viewed under UV fluorescence with appropriate filters (Olympus).

Centromeric, BAC, and fosmid probes: Mitotic chromosome spreads were prepared as described above. Slides were treated with RNase at 100 ug/ml for 1 h at 37°C and washed in 2xSSC and dehydrated in 70%, 90% and 100% ethanol. Chromosomal DNA was denatured at 75°C for 3 minutes in 70% formamaide/2XSSC, followed by dehydration in ice cold 70%, 90% and 100% ethanol. BAC and Fosmid DNAs were nick-translated using standard protocols to incorporate biotin-11-dUTP or digoxigenin-dUTP (Invitrogen). BAC and Fosmid DNAs were directly labeled with Cy3-dUTP, FITC-dUTP, Spectrum Orange-dUTP or Spectrum Green_dUTP (Vysis, Abbott Laboratories) using nick-translation or random priming using standard protocols. Final probe concentrations varied from 40–60 ng/*µ*l. Centromeric probe cocktails (Vysis) plus BAC or Fosmid DNAs were denatured at 75°C for 10 minutes and prehybridized at 37°C for 30 minutes. Probes were applied to slides and incubated overnight at 37°C. Post-hybridization washes consisted of three 3-minute rinses in 50% formamide/2XSSC, three 3-minute rinses in 2XSSC, and finally three 3-minute rinses in PN buffer (0.1M Na2HPO4 + 0.0M NaH2PO4, ph 8.0, +2.5% Nonidet NP-40), all at 45°C. Signal detection was carried out as described [61]. Amplification of biotinylated probe signal utilized alternating incubations of slides with anti-avidin (Vector) and FITC-Extravidin (Sigma). Slides were then counterstained with either propidium iodide (2.5 ug/ml) or DAPI (15 ug/ml) and viewed under UV fluorescence (Olympus).

### RNA-DNA FISH

The methods for the RNA-DNA FISH assays in this study were carried out as previously described [18]. Cells were plated on microscope slides treated with concanavalin A (Sigma) at ∼50% confluence and incubated for 4 hours in complete media in a 37°C humidified CO_2_ incubator. Slides were rinsed 1 time with sterile RNase free PBS. Slides were incubated for 30 seconds in CSK buffer (100 mM NaCl, 300 mM Sucrose, 3 mM MgCl_2,_10 mM Pipes, ph 6.8), 5 minutes in CSK buffer plus 0.1% Triton X-100, and then for an addition 30 seconds in CSK buffer at room temperature. Cells were fixed in 4% paraformaldehyde in PBS for 10 minutes at room temperature. Slides were rinsed in 70% ETOH and stored in 70% ETOH at 4°C until use. Just prior to use, slides were dehydrated through an ETOH series (70%, 90% and 100%) and allowed to air dry. Denatured probes were prehybridized with Cot-1 DNA at 37°C for 30 min. Slides were hybridized at 37°C for 14–16 hours. Slides were washed as follows: 3 times in 50% formamide/2xSSC at 42°C for 5 minutes, 3 times in 2xSSC at 42°C for 5 minutes, 3 times in 4xSSC/0.1% Tween 20 at room temperature for 3 minutes. Slides were then counterstained with either propidium iodide (2.5 ug/ml) or DAPI (15 ug/ml) and viewed under UV fluorescence (Olympus). Z-stack images were generated using a Cytovision workstation. The hybridization signals on individual cells were captured and the slide coordinates recorded. The slides were then dehydrated in 70%, 90% and 100% ETOH, and then processed for DNA FISH, including the RNAase treatment step, as described above. The same cells that were imaged with the RNA FISH probes were located on the slides and then re-imaged for the DNA hybridization signals.

### Replication timing assay

The methods for the replication timing assays in this study were carried out as previously described [19], [23]. The BrdU replication-timing assay was performed on exponentially dividing cultures as follows: asynchronously growing cells were exposed to 20 ug/ml of BrdU (Sigma) for 4.5 or 6 hours. Mitotic cells were harvested in the absence of colcemid, treated with 75 mM KCl for 15–30 minutes at 37°C, fixed in 3∶1 methanol∶acetic acid and dropped on wet ice cold slides. The chromosomes were denatured in 70% formamide in 2xSSC at 70°C for 3 minutes and processed for DNA FISH, as described above. The incorporated BrdU was then detected using a FITC-labeled anti-BrdU antibody (Becton Dickinson). Slides were stained with propidium iodide (0.3 mg/ml), cover slipped, and viewed under UV fluorescence.

All images were captured with an Olympus BX Fluorescent Microscope using a 100X objective, automatic filter-wheel and Cytovision workstation. Individual chromosomes were identified with either chromosome-specific paints or centromeric probes in combination with BACs from the deleted regions (see DNA FISH procedure above). Utilizing the Cytovision workstation, each chromosome was isolated from the metaphase spread and a line drawn along the middle of the entire length of the chromosome. The Cytovision software was used to calculate the pixel area and intensity along each chromosome for each fluorochrome occupied by the DAPI and BrdU (FITC) signals. The total amount of fluorescent signal was calculated by multiplying the average pixel intensity by the area occupied by those pixels.

### ReTiSH

We used the ReTiSH assay essentially as described [34]. Briefly, unsynchronized, exponentially growing cells were treated with 30 µM BrdU (Sigma) for 6 or 5 and 14 hours. Colcemid (Sigma) was added to a final concentration of 0.1 µg/mL for 1 h at 37°C. Cells were trypsinized, centrifuged at 1,000 rpm, and resuspended in prewarmed hypotonic KCl solution (0.075 M) for 40 min at 37°C. Cells were pelleted by centrifugation and fixed with methanol-glacial acetic acid (3∶1). Fixed cells were drop gently onto wet, cold slides and allowed to air-dry. Slides were treated with 100 µg/ml RNAse A at 37°C for 10 min. Slides were rinsed briefly in d_2_H_2_0 followed by fixation in 4% formaldehyde at room temperature for 10 minutes. Slides were incubated with pepsin (1 mg/mL in 2N HCl) for 10 min at 37°C, and then rinsed again with d_2_H_2_0 and stained with 0.5 µg/µL Hoechst 33258 (Sigma) for 15 minutes. Slides were flooded with 200 µl 2xSSC, coversliped and exposed to 365-nm UV light for 30 min using a UV Stratalinker 2400 transilluminator (Stratagene). Slides were rinsed with d_2_H_2_0 and drained. Slides were incubated with 100 µl of 3U/µl of ExoIII (Fermentas) in ExoIII buffer for 15 min at 37°C. The slides were then processed directly for DNA FISH as described above, except with the absence of a denaturation step.

## Supporting Information

S1 Fig Southern blot hybridizations showing similar and independent Lentiviral integrations in clones from different pools of Lentiviral infected P268 cells. The top panel shows a blot hybridized with the 3′ half (RT) of mouse *Aprt* as probe (top panel), and the bottom panel shows a blot hybridized with the 5′ half (AP) of mouse *Aprt* as probe. Genomic DNA form independent clones (lowercase letters), generated from three different pools (15,17, and 18) of P268 cells infected with a Lentivirus containing the AP-loxP cassette, were isolated following transient Cre expression and Aprt selection are shown. Genomic DNA was digested with Bcl1, and the size markers (M) in kb are shown. Genomic DNA from P268 and R268 were used as controls. Note that the ∼15 kb RT band present in P268 (top panel) is shifted in all of the deletion clones, indicating a Cre-mediated recombination event involving the original RT-loxP integration site. Also note that the new RT bands are different in size than in R268, indicating that the t (15;16) was not generated and therefore each clone contains a rearrangement with the Lentiviral loxP site. The bottom panel (AP probe) shows the original AP-loxP bad in P268 DNA and new AP bands corresponding to the Lentiviral AP-loxP cassettes in the deletion clones.
(TIF)

S2 Fig A) Schematic view of the original loxP-RT integration site in P268 cells. The integration site was determined by inverse PCR and is located at 76,858,743. The approximate location of the cassette-genome junction-PCR primers (green half arrows) is indicated for both the proximal and distal junctions. B) Junction PCR for the detection of deletions. Individual Aprt+ clones isolated from the indicated pools (#17 and #18) were subjected to PCR reactions with the proximal and distal junction-PCR primers (see panel A). Note that all of the clones have lost the distal junction but retain the proximal junction. Genomic DNA from P268 was used as positive control, and DNA from P175, which contains a loxP-RT insertion in chromosome 6 [19] and should not contain either chromosome 15 junction, was used as a negative control. C) Junction PCR for the Lentiviral-genome junctions. Integration sites were determined by LAM-PCR and primers directed to the integration site were used in combination with a primer to Lentiviral 5′ LTR sequence. Genomic DNAs isolated from individual Aprt+ clones, from Pool #4, were subjected to PCR reactions with genome-Lentiviral junction-PCR primers. Note that clones a, e, g, and m resulted in a PCR product and therefore contain the same Lentiviral integration site. D) LOH analysis in cells with a Cre-loxP deletion in chromosome 15. Sequencing traces from PCR products generated from P268 and Δ268-4d cells are shown. The arrows mark the location of the heterozygous SNP (rs2881582).
(TIF)

S3 Fig Replication timing assay on chromosome 15 with an ∼124 kb distal deletion. A-F) Δ268-18a cells were incubated with BrdU for 6 hours, harvested for mitotic cells, stained with an antibody to BrdU (green) and processed for DNA FISH using a chromosome 15 centromeric probe (red) plus BAC CTD-2299E17 (red). The chromosomal DNA was stained with DAPI (blue). A and C) Three chromosome 15 s (i, ii, and iii) from single metaphase cells are shown in each panel. Chromosomes i in each panel contain the ∼124 kb deletion. The three chromosome 15 s were cut out and aligned showing the BrdU and FISH signals in separate images. The asterisks mark the location of the deletion in the chromosomes marked i, and the arrows mark the location of the BAC hybridization signals on chromosomes ii and iii. B and D) Pixel intensity profiles of the BrdU incorporation (green), and DAPI (blue) staining along the three chromosome 15 s from panel A and C, respectively. E) Quantification of the BrdU incorporation in multiple cells. The red and blue bars represent deleted and non-deleted chromosome 15 s, respectively. The total pixels (average intensity x area) for each chromosome showing the amount of BrdU incorporation are shown. F) Secondary rearrangements of chromosome 15 containing the ∼124 kb deletion in chromosome 15. Mitotic cells from Δ268-18a were processed for DNA FISH with a chromosome 15 WCP, and the chromosomal DNA was stained with DAPI. Rearrangements involving chromosome 15 are indicated with arrows, and non-rearranged chromosome 15 s are indicated with asterisks.
(TIF)

S4 Fig Chromosome 15 replication timing assay in P268 cells. A-F) P268 cells were incubated with BrdU for 6 hours, harvested for mitotic cells, stained with an antibody to BrdU (green) and processed for DNA FISH using a chromosome 15 WCP as probe (red). The chromosomal DNA was stained with DAPI (blue). A and B) A metaphase spread containing three chromosome 15 s (i, ii, and iii). C) The three chromosome 15 s from panel B were cut out and aligned showing the BrdU and FISH signals in separate images. D) Pixel intensity profiles of the BrdU incorporation (green), and DAPI (blue) staining along the three chromosome 15 s from panel B. E) The pixel intensity (average intensity x area) for each chromosome, i, ii, and iii, showing the total amount of BrdU incorporation or DAPI staining. F) Quantification of the BrdU incorporation in multiple cells. The bars represent different chromosome 15 s in 7 different cells.
(TIF)

S5 Fig Replication timing assay on chromosome 15 with an ∼2 kb distal deletion. A-F) Δ268-15c cells were incubated with BrdU for 6 hours, harvested for mitotic cells, stained with an antibody to BrdU (green) and processed for DNA FISH using a chromosome 15 WCP as probe (red). The chromosomal DNA was stained with DAPI (blue). A and B) A metaphase spread containing four chromosome 15 s (i, ii, iii and iv). C) The four chromosome 15 s from panel B were cut out and aligned showing the BrdU and FISH signals in separate images. D) Pixel intensity profiles of the BrdU incorporation (green), and DAPI (blue) staining along the four chromosome 15 s from panel B. E) The pixel intensity (average intensity x area) for each chromosome, i, ii, iii, and iv showing the total amount of BrdU incorporation or DAPI staining. F) Quantification of the BrdU incorporation in multiple cells. The bars represent different chromosome 15 s in 5 different cells.
(TIF)

S6 Fig Replication timing assay on chromosome 15 with an ∼67–126 kb proximal deletion. A–D) Δ268F-6b cells were incubated with BrdU for 6 hours, harvested for mitotic cells, stained with an antibody to BrdU (green) and processed for DNA FISH using a chromosome 15 centromeric probe (red) plus BAC CTD-2117F7 (red). The chromosomal DNA was stained with DAPI (blue). A) Three chromosome 15 s from a metaphase spread were cut out and aligned showing the BrdU and FISH signals in separate images. The asterisk marks the location of the deletion in the chromosome marked i, and the arrows mark the location of the BAC hybridization signals on chromosomes ii and iii. B) Pixel intensity profiles of the BrdU incorporation (green), and DAPI (blue) staining along the three chromosome 15 s from panel A. C) The pixel intensities (average intensity x area) for each chromosome, i, ii, and iii, showing the total amount of BrdU incorporation or DAPI staining. D) Quantification of the BrdU incorporation in multiple cells. The red and blue bars represent deleted and non-deleted chromosome 15 s, respectively, in 6 different cells. E) Lack of rearrangements of chromosome 15 containing in Δ268F-6b cells. Mitotic cells were processed for DNA FISH with a chromosome 15 WCP, and the chromosomal DNA was stained with DAPI. F) Quantification of the BrdU incorporation in multiple cells with the ∼18 mb proximal deletion. Δ268F-6a cells were processed as in panels A–D. The red and blue bars represent deleted and non-deleted chromosome 15 s, respectively, in 7 different cells. G) Lack of rearrangements of chromosome 15 containing an ∼18 mb proximal deletion in Δ268F-6a cells. Mitotic cells were processed for DNA FISH with a chromosome 15 WCP. E and G) The asterisks mark non-rearranged chromosome 15 s, and the chromosomal DNA was stained with DAPI.
(TIF)

S7 Fig Delayed replication of chromosome 15 s with inversions. A–B) Inv268-6c cells were incubated with BrdU for 6 hours, harvested for mitotic cells, stained with an antibody to BrdU (green) and processed for DNA FISH using a chromosome 15 WCP as probe (red). The chromosomal DNA was stained with DAPI (blue). A) Three chromosome 15 s from a metaphase cells were cut out and aligned showing the BrdU and FISH signals in separate images. The asterisk marks a chromosome with delayed replication. B) Secondary rearrangements of chromosome 15 in cells containing an inversion in chromosome 15. Mitotic cells from Inv268-6c were processed for DNA FISH with a chromosome 15 WCP, and the chromosomal DNA was stained with DAPI. Rearrangements involving chromosome 15 are indicated with arrows, and non-rearranged chromosome 15 s are indicated with asterisks. C) Three chromosome 15 s from a metaphase cells from Inv268-3c cells were cut out and aligned showing the BrdU and FISH signals in separate images. The asterisk marks a chromosome with delayed replication. D) Secondary rearrangements of chromosome 15 containing an inversion in chromosome 15. Mitotic cells from Inv268-3c were processed for DNA FISH with a chromosome 15 WCP, and the chromosomal DNA was stained with DAPI. Rearrangements involving chromosome 15 are indicated with arrows, and non-rearranged chromosome 15 s are indicated with asterisks.
(TIF)

S8 Fig RNA and DNA FISH using fosmid probes A4 and F8 in combination with E4. A) P268 cells were subjected to RNA FISH using the E4 (green) and A4 (red) probes to detect RNA simultaneously. Representative images from three different P268 cells (#1-3) are shown in each panel. The arrows mark the sites of E4 hybridization. B) DNA FISH using E4 (green) and A4 (red) probes to detect DNA simultaneously on metaphase spreads from Δ268F-4t cells, which contain an ∼135 kb deletion that includes the DNA represented by the E4 fosmid but does not delete the A4 fosmid (see Fig. 5A). The arrows mark the sites of hybridization to the E4 plus A4 probes, and the arrow with the asterisk marks the chromosome 15 that only hybridized to the A4 probe. We detected hybridization of the A4 probe to 100% of chromosome 15 s that hybridized to the E4 probe, plus one site of hybridization of the A4 probe to a chromosome 15 without the E4 signal, which allowed us to identify the deleted chromosome 15 s. C) P268 cells were subjected to RNA FISH using the E4 (green) and F8 (red) probes to detect RNA simultaneously. Representative images from three different P268 cells (#1-3) are shown in each panel. The arrows mark the sites of E4 hybridization. D) DNA FISH using E4 (green) and F8 (red) probes to detect DNA simultaneously on metaphase spreads from Δ268F-4t cells, which contain an ∼135 kb deletion that includes the DNA represented by the E4 fosmid but does not delete the F8 fosmid (see Fig. 5A). The arrows mark the sites of hybridization to the E4 plus F8 probes, and the arrow with the asterisk marks the chromosome 15 that only hybridized to the F8 probe. We detected hybridization of the F8 probe to 100% of chromosome 15 s that hybridized to the E4 probe, plus one site of hybridization of the F8 probe to a chromosome 15 without the E4 signal, which allowed us to identify the deleted chromosome 15 s.
(TIF)

S9 Fig RNA-DNA FISH in HepG2 cells. Human HepG2 cells were processed for RNA-DNA FISH using the H1 probe to detect RNA plus a BAC from the *ASAR15* locus to detect DNA. Slides were subjected to RNA FISH using the H1 fosmid (green) to detect RNA. Slides were subsequently processed for DNA FISH using a BAC CTD-2299E17 to detect DNA. The nuclear DNA was stained with DAPI. A–C) A representative field of cells showing the RNA FISH (A), DNA FISH (B) and merged images (C). D) Representative cells (#1 and #2) with large “clouds” of hybridization are shown. The arrows mark the location of the DNA signals that have a corresponding RNA signal, and the arrowheads mark the location of the DNA signals that lack a corresponding RNA signal. The nuclear DNA was stained with DAPI. E and F). RNA FISH in female HDFs. Human female HDF cells were processed for RNA FISH using the H1 *ASAR15* probe (green) in combination with an XIST (red) probe. The arrows mark the large clouds of RNA detected by the *XIST* probe and the arrowheads mark the variably sized hybridization signals detected by the *ASAR15* probe. The DNA was stained with DAPI.
(TIF)

S10 Fig RNAseq data from HepG2 cells using the UCSC Genome Browser. The genomic region between 76,825,000 and 77,015,000 bp of human chromosome 15 (NCBI Build 37/hg19) illustrating SCAPER intron-exon junctions, MIR3713, the location of the original loxP-RT integration site in P268 cells, and the location of the AP-loxP integration site for the ∼135 kb deletion. Two different screenshots, separated by a doted line, show the proximal and distal halves of this region of chromosome 15. Also shown is RNA-seq data, cytosol poly A-, cytosol poly A+, nucleus poly A-, and nucleus poly A+, from the cell line HepG2. The blue tick marks indicate sequence hits from the plus direction, and the red tick marks indicate sequence hits from the minus direction. Note that RNA in the SCPAER introns is enriched in the poly A- fraction in the nucleus, and that there is very little plus strand RNA synthesized, including in the region encoding MIR3713, which is transcribed from the plus strand.
(TIF)

S11 Fig *ASAR15* is transcribed by RNA Polymerase II and has a relatively long half-life. Cells were exposed to 20 ug/mL of α-amanitin for 0, 5, and 10 hours. Total RNA was subjected to reverse transcriptase reactions (RT) in the presence (+) or absence (-) of reverse transcriptase followed by semi-quantitative PCR using primers to a tRNA gene (RNA Pol III), 45S RNA (RNA Pol I), *P300* cDNA (RNA Pol II), *SCAPER* cDNA (spanning exons 4 and 5; RNA Pol II) *ASAR6* (RNA Pol II), and primers from within SCAPER introns 21 (rs11072602), 23 (rs12916573), and 24 (rs59088060). DNA size ladder was used for size reference (M).
(TIF)

S1 Table Summary of the Cre/loxP-mediated chromosome rearrangements analyzed. The top panel (Deletion Clones) indicates the name of each clone (Δ268-) and the size of the proximal or distal deletions generated from the original loxP-RT integration site in chromosome 15 in P268 cells. The middle panel (Inversion Clones) indicates the name of each clone (Inv268-) and the size of the proximal or distal inversions generated from the original loxP-RT integration site in chromosome 15 in P268 cells. The bottom panel (Aprt transfected Clones) indicates the name of each control P268 clone, generated by transfection of an Aprt expression vector, selection in media containing Azaserine and adenine, and subsequent clonal expansion, analyzed. The number of chromosome rearrangements detected using FISH with a chromosome 15 whole chromosome paint (CHR 15 Paint), and the total number of cells scored are indicated. Also shown are the replication-timing assays (Brdu-WCP and BrdU-BAC) used and whether or not delayed replication timing (DRT) was detected for each clone in each panel.
(PDF)
