# Supplementary figures and images for: ASAR15, A cis-Acting Locus that Controls Chromosome-Wide Replication Timing and Stability of Human Chromosome 15
Source: PLoS Genet. 2015 Jan 8;11(1):e1004923. doi: 10.1371/journal.pgen.1004923 (PMC4287527; doi:10.1371/journal.pgen.1004923)

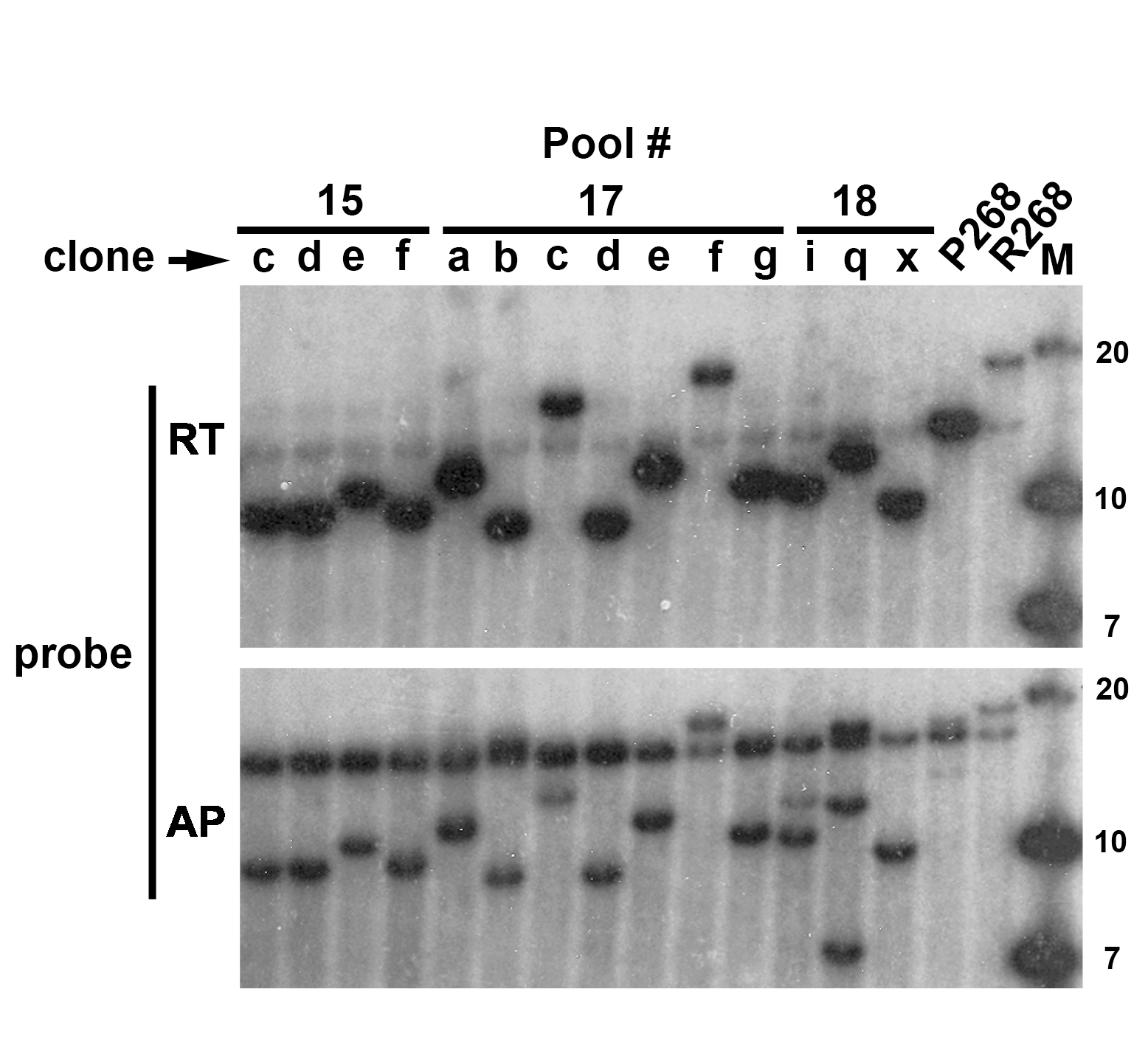

Supplement: S1 Fig — Southern blot hybridizations showing similar and independent Lentiviral integrations in clones from different pools of Lentiviral infected P268 cells. The top panel shows a blot hybridized with the 3′ half (RT) of mouse Aprt as probe (top panel), and the bottom panel shows a blot hybridized with the 5′ half (AP) of mouse Aprt as probe. Genomic DNA form independent clones (lowercase letters), generated from three different pools (15,17, and 18) of P268 cells infected with a Lentivirus containing the AP-loxP cassette, were isolated following transient Cre expression and Aprt selection are shown. Genomic DNA was digested with Bcl1, and the size markers (M) in kb are shown. Genomic DNA from P268 and R268 were used as controls. Note that the ∼15 kb RT band present in P268 (top panel) is shifted in all of the deletion clones, indicating a Cre-mediated recombination event involving the original RT-loxP integration site. Also note that the new RT bands are different in size than in R268, indicating that the t (15;16) was not generated and therefore each clone contains a rearrangement with the Lentiviral loxP site. The bottom panel (AP probe) shows the original AP-loxP bad in P268 DNA and new AP bands corresponding to the Lentiviral AP-loxP cassettes in the deletion clones. (TIF) [file pgen.1004923.s001.tif]

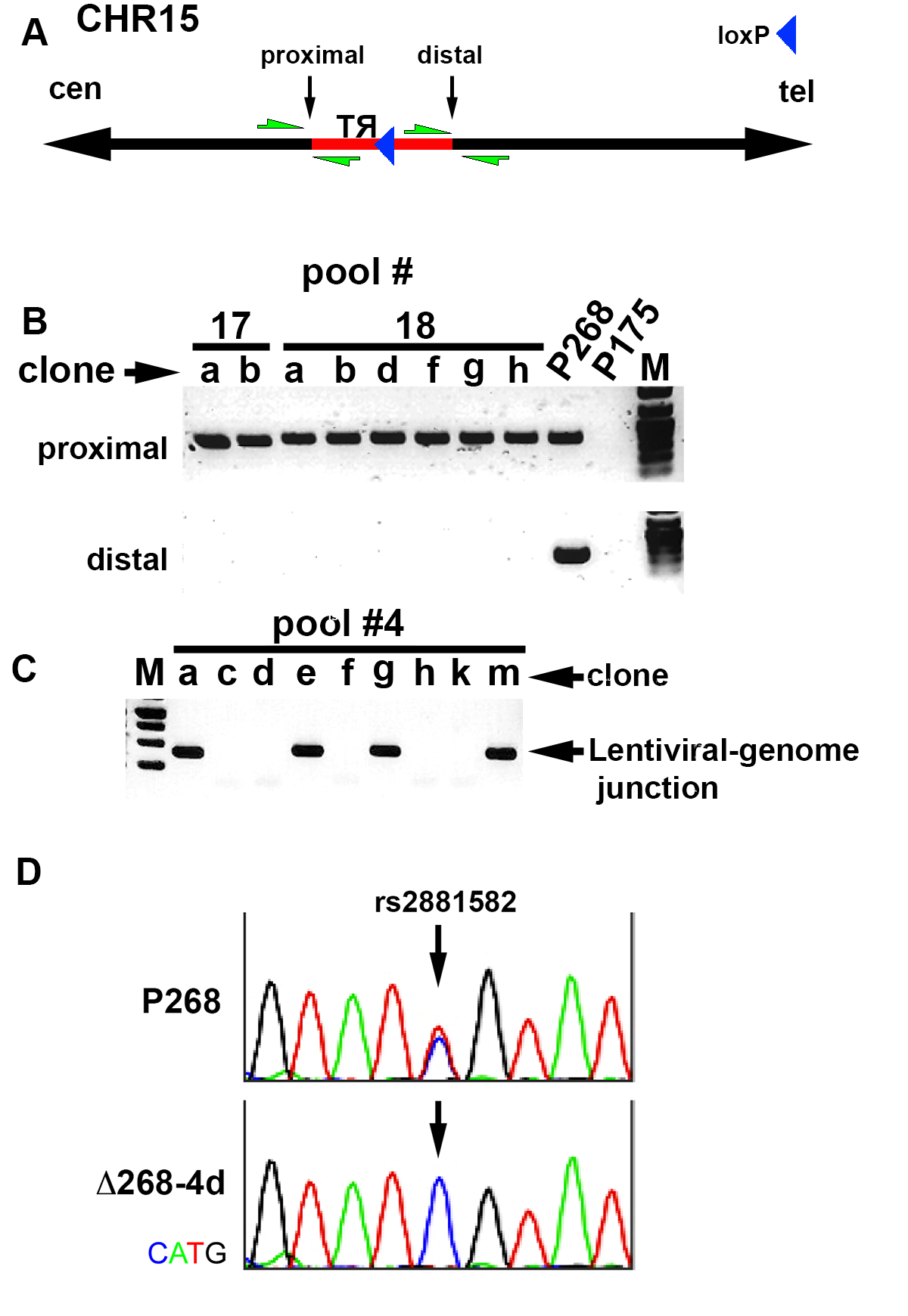

Supplement: S2 Fig — A) Schematic view of the original loxP-RT integration site in P268 cells. The integration site was determined by inverse PCR and is located at 76,858,743. The approximate location of the cassette-genome junction-PCR primers (green half arrows) is indicated for both the proximal and distal junctions. B) Junction PCR for the detection of deletions. Individual Aprt+ clones isolated from the indicated pools (#17 and #18) were subjected to PCR reactions with the proximal and distal junction-PCR primers (see panel A). Note that all of the clones have lost the distal junction but retain the proximal junction. Genomic DNA from P268 was used as positive control, and DNA from P175, which contains a loxP-RT insertion in chromosome 6 [19] and should not contain either chromosome 15 junction, was used as a negative control. C) Junction PCR for the Lentiviral-genome junctions. Integration sites were determined by LAM-PCR and primers directed to the integration site were used in combination with a primer to Lentiviral 5′ LTR sequence. Genomic DNAs isolated from individual Aprt+ clones, from Pool #4, were subjected to PCR reactions with genome-Lentiviral junction-PCR primers. Note that clones a, e, g, and m resulted in a PCR product and therefore contain the same Lentiviral integration site. D) LOH analysis in cells with a Cre-loxP deletion in chromosome 15. Sequencing traces from PCR products generated from P268 and Δ268-4d cells are shown. The arrows mark the location of the heterozygous SNP (rs2881582). (TIF) [file pgen.1004923.s002.tif]

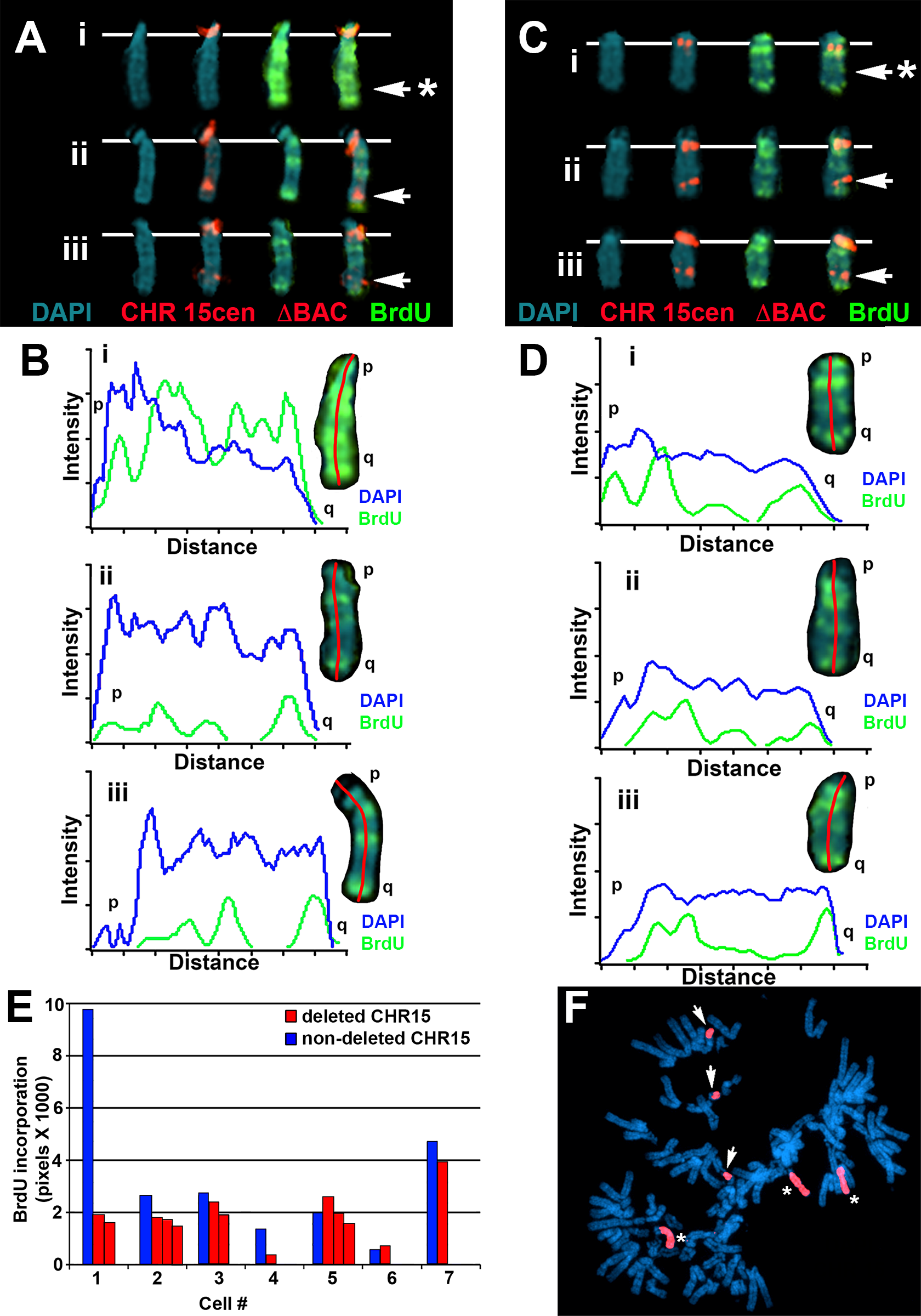

Supplement: S3 Fig — Replication timing assay on chromosome 15 with an ∼124 kb distal deletion. A-F) Δ268-18a cells were incubated with BrdU for 6 hours, harvested for mitotic cells, stained with an antibody to BrdU (green) and processed for DNA FISH using a chromosome 15 centromeric probe (red) plus BAC CTD-2299E17 (red). The chromosomal DNA was stained with DAPI (blue). A and C) Three chromosome 15 s (i, ii, and iii) from single metaphase cells are shown in each panel. Chromosomes i in each panel contain the ∼124 kb deletion. The three chromosome 15 s were cut out and aligned showing the BrdU and FISH signals in separate images. The asterisks mark the location of the deletion in the chromosomes marked i, and the arrows mark the location of the BAC hybridization signals on chromosomes ii and iii. B and D) Pixel intensity profiles of the BrdU incorporation (green), and DAPI (blue) staining along the three chromosome 15 s from panel A and C, respectively. E) Quantification of the BrdU incorporation in multiple cells. The red and blue bars represent deleted and non-deleted chromosome 15 s, respectively. The total pixels (average intensity x area) for each chromosome showing the amount of BrdU incorporation are shown. F) Secondary rearrangements of chromosome 15 containing the ∼124 kb deletion in chromosome 15. Mitotic cells from Δ268-18a were processed for DNA FISH with a chromosome 15 WCP, and the chromosomal DNA was stained with DAPI. Rearrangements involving chromosome 15 are indicated with arrows, and non-rearranged chromosome 15 s are indicated with asterisks. (TIF) [file pgen.1004923.s003.tif]

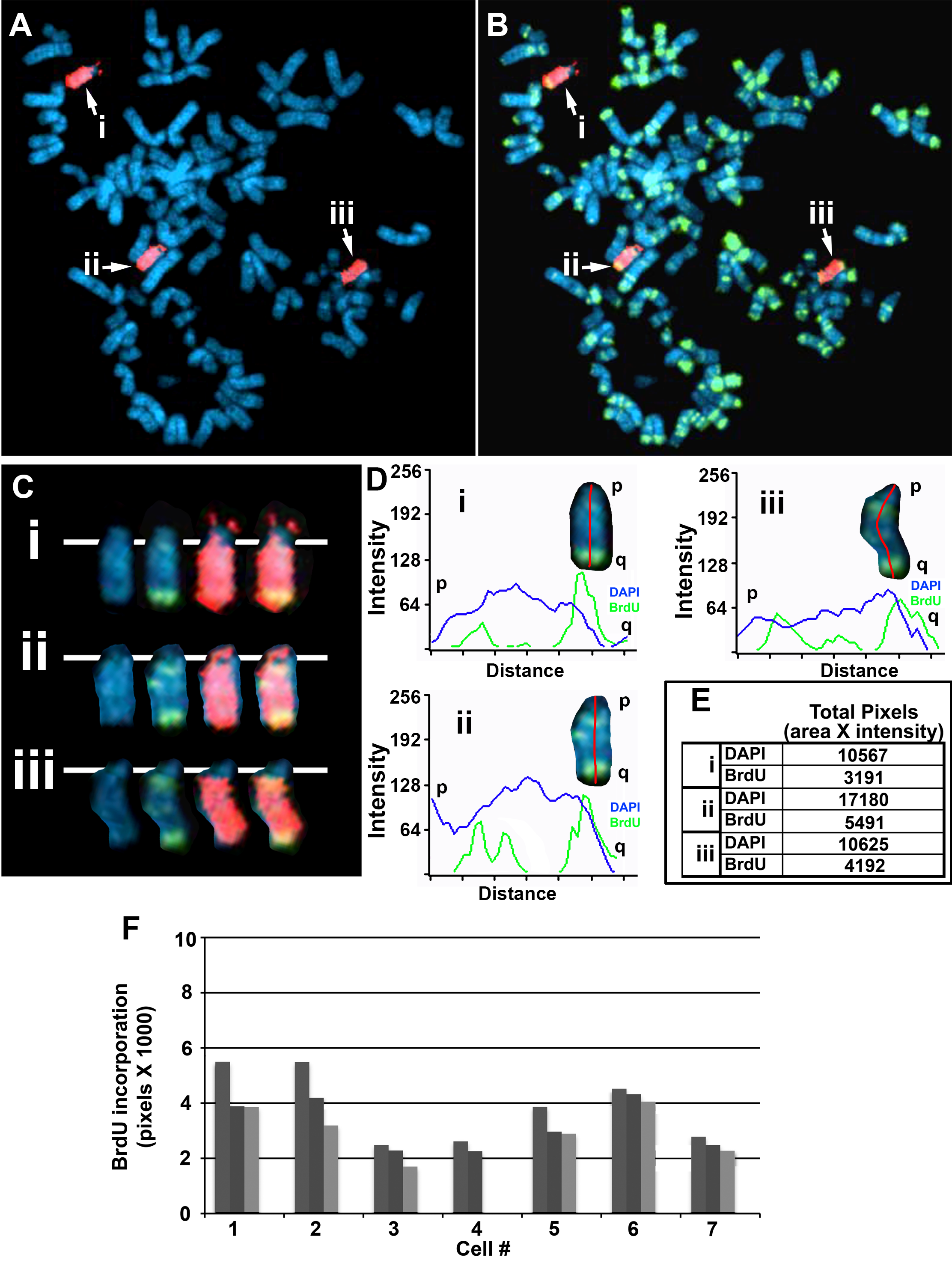

Supplement: S4 Fig — Chromosome 15 replication timing assay in P268 cells. A-F) P268 cells were incubated with BrdU for 6 hours, harvested for mitotic cells, stained with an antibody to BrdU (green) and processed for DNA FISH using a chromosome 15 WCP as probe (red). The chromosomal DNA was stained with DAPI (blue). A and B) A metaphase spread containing three chromosome 15 s (i, ii, and iii). C) The three chromosome 15 s from panel B were cut out and aligned showing the BrdU and FISH signals in separate images. D) Pixel intensity profiles of the BrdU incorporation (green), and DAPI (blue) staining along the three chromosome 15 s from panel B. E) The pixel intensity (average intensity x area) for each chromosome, i, ii, and iii, showing the total amount of BrdU incorporation or DAPI staining. F) Quantification of the BrdU incorporation in multiple cells. The bars represent different chromosome 15 s in 7 different cells. (TIF) [file pgen.1004923.s004.tif]

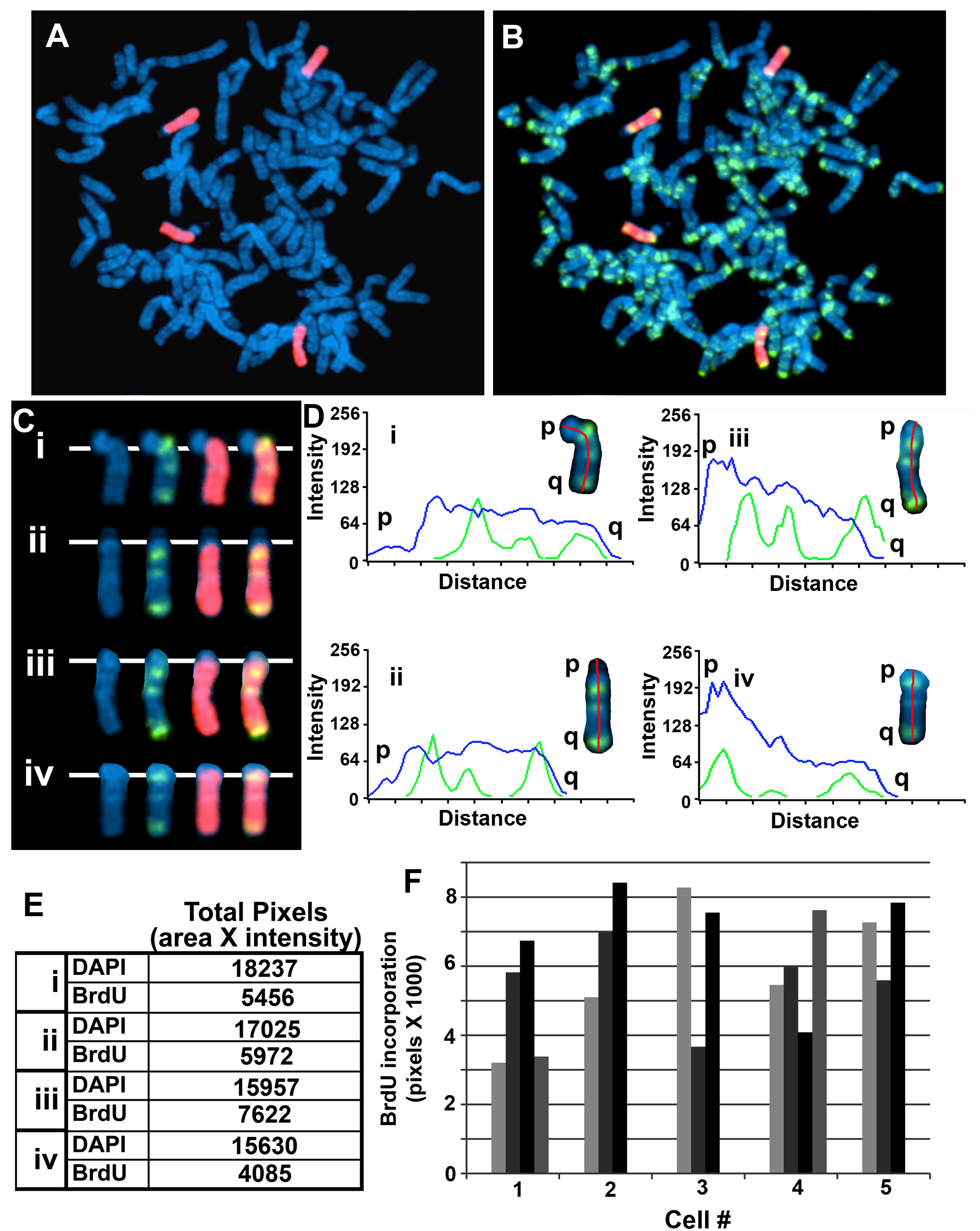

Supplement: S5 Fig — Replication timing assay on chromosome 15 with an ∼2 kb distal deletion. A-F) Δ268-15c cells were incubated with BrdU for 6 hours, harvested for mitotic cells, stained with an antibody to BrdU (green) and processed for DNA FISH using a chromosome 15 WCP as probe (red). The chromosomal DNA was stained with DAPI (blue). A and B) A metaphase spread containing four chromosome 15 s (i, ii, iii and iv). C) The four chromosome 15 s from panel B were cut out and aligned showing the BrdU and FISH signals in separate images. D) Pixel intensity profiles of the BrdU incorporation (green), and DAPI (blue) staining along the four chromosome 15 s from panel B. E) The pixel intensity (average intensity x area) for each chromosome, i, ii, iii, and iv showing the total amount of BrdU incorporation or DAPI staining. F) Quantification of the BrdU incorporation in multiple cells. The bars represent different chromosome 15 s in 5 different cells. (TIF) [file pgen.1004923.s005.tif]

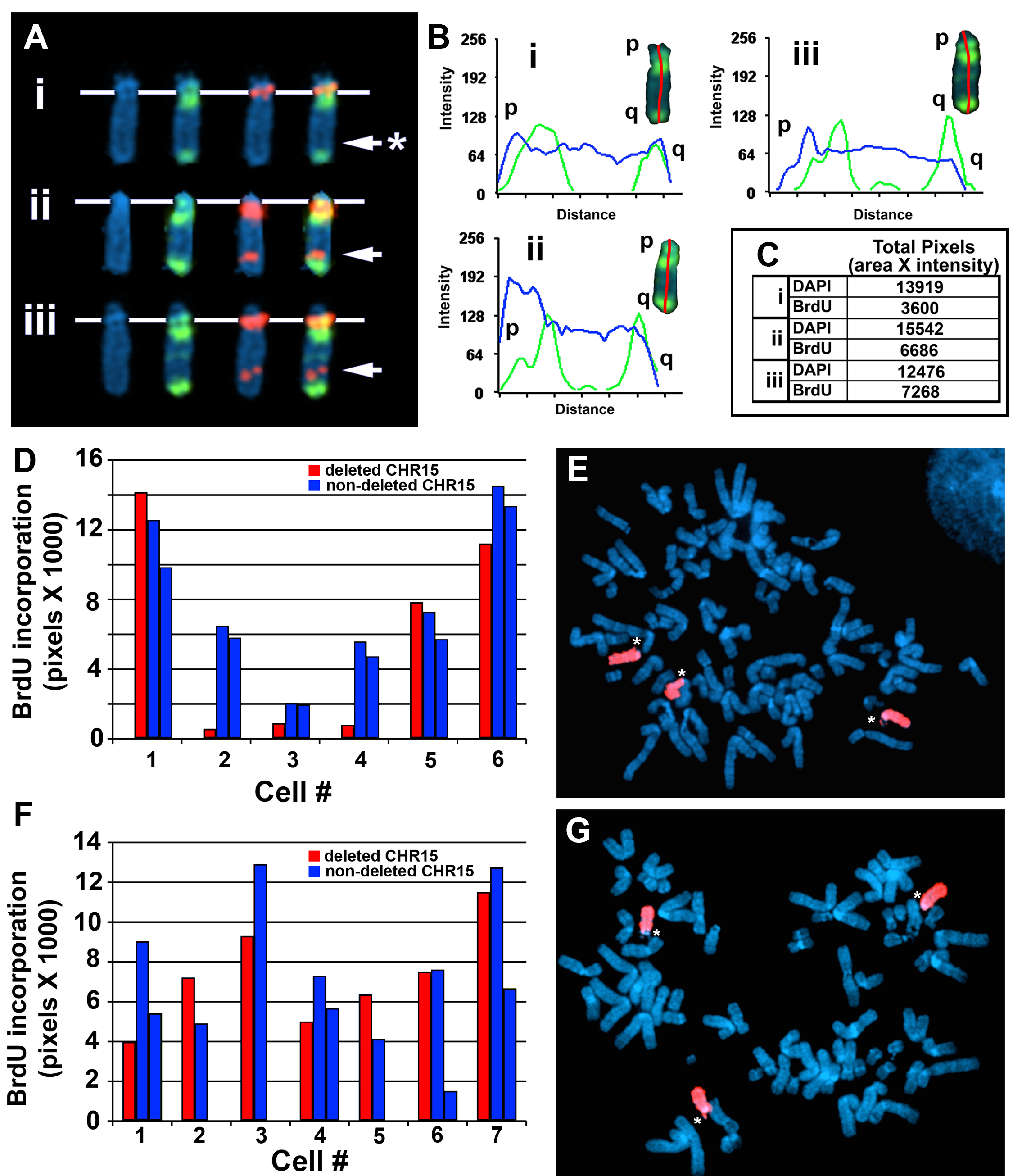

Supplement: S6 Fig — Replication timing assay on chromosome 15 with an ∼67–126 kb proximal deletion. A–D) Δ268F-6b cells were incubated with BrdU for 6 hours, harvested for mitotic cells, stained with an antibody to BrdU (green) and processed for DNA FISH using a chromosome 15 centromeric probe (red) plus BAC CTD-2117F7 (red). The chromosomal DNA was stained with DAPI (blue). A) Three chromosome 15 s from a metaphase spread were cut out and aligned showing the BrdU and FISH signals in separate images. The asterisk marks the location of the deletion in the chromosome marked i, and the arrows mark the location of the BAC hybridization signals on chromosomes ii and iii. B) Pixel intensity profiles of the BrdU incorporation (green), and DAPI (blue) staining along the three chromosome 15 s from panel A. C) The pixel intensities (average intensity x area) for each chromosome, i, ii, and iii, showing the total amount of BrdU incorporation or DAPI staining. D) Quantification of the BrdU incorporation in multiple cells. The red and blue bars represent deleted and non-deleted chromosome 15 s, respectively, in 6 different cells. E) Lack of rearrangements of chromosome 15 containing in Δ268F-6b cells. Mitotic cells were processed for DNA FISH with a chromosome 15 WCP, and the chromosomal DNA was stained with DAPI. F) Quantification of the BrdU incorporation in multiple cells with the ∼18 mb proximal deletion. Δ268F-6a cells were processed as in panels A–D. The red and blue bars represent deleted and non-deleted chromosome 15 s, respectively, in 7 different cells. G) Lack of rearrangements of chromosome 15 containing an ∼18 mb proximal deletion in Δ268F-6a cells. Mitotic cells were processed for DNA FISH with a chromosome 15 WCP. E and G) The asterisks mark non-rearranged chromosome 15 s, and the chromosomal DNA was stained with DAPI. (TIF) [file pgen.1004923.s006.tif]

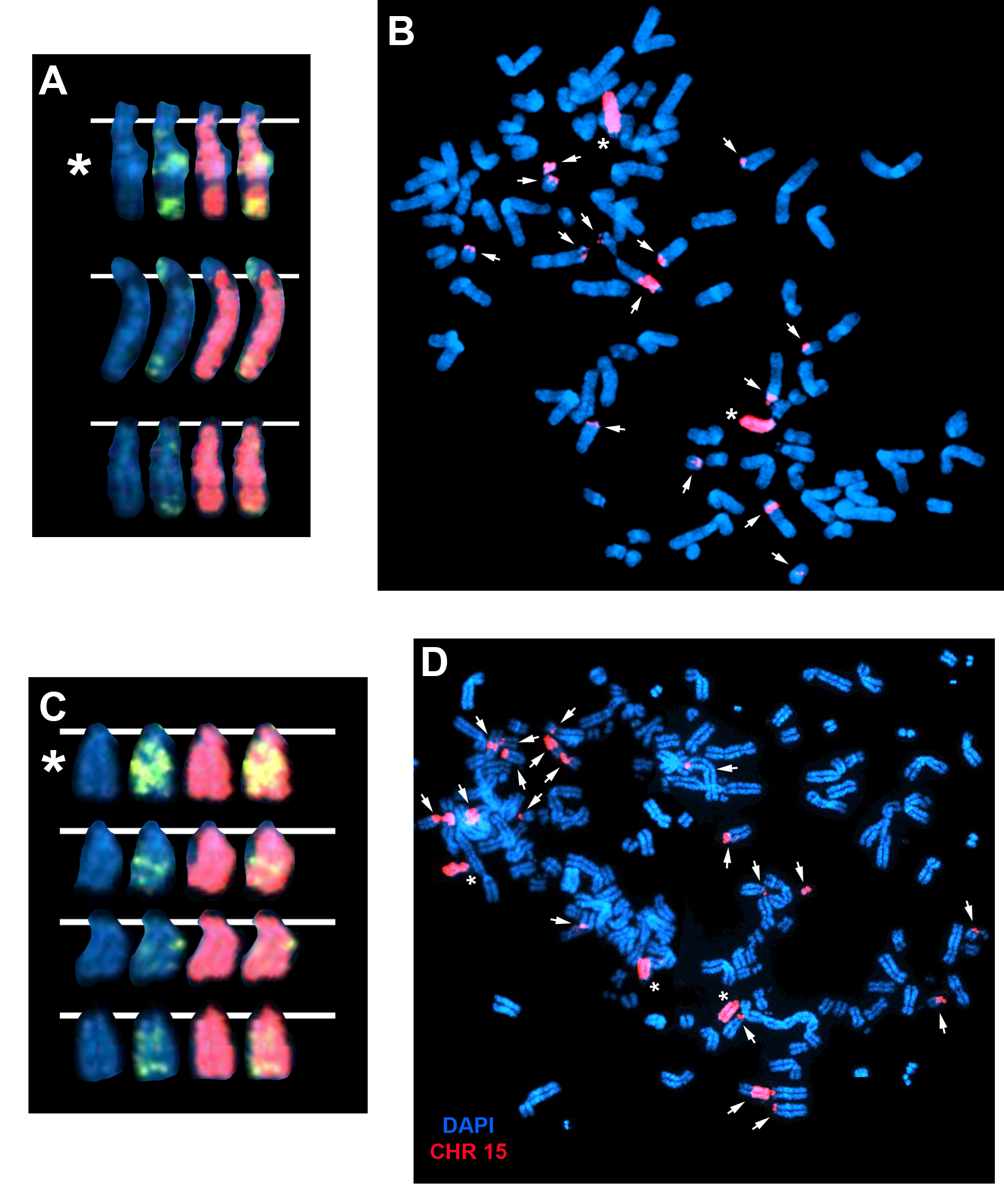

Supplement: S7 Fig — Delayed replication of chromosome 15 s with inversions. A–B) Inv268-6c cells were incubated with BrdU for 6 hours, harvested for mitotic cells, stained with an antibody to BrdU (green) and processed for DNA FISH using a chromosome 15 WCP as probe (red). The chromosomal DNA was stained with DAPI (blue). A) Three chromosome 15 s from a metaphase cells were cut out and aligned showing the BrdU and FISH signals in separate images. The asterisk marks a chromosome with delayed replication. B) Secondary rearrangements of chromosome 15 in cells containing an inversion in chromosome 15. Mitotic cells from Inv268-6c were processed for DNA FISH with a chromosome 15 WCP, and the chromosomal DNA was stained with DAPI. Rearrangements involving chromosome 15 are indicated with arrows, and non-rearranged chromosome 15 s are indicated with asterisks. C) Three chromosome 15 s from a metaphase cells from Inv268-3c cells were cut out and aligned showing the BrdU and FISH signals in separate images. The asterisk marks a chromosome with delayed replication. D) Secondary rearrangements of chromosome 15 containing an inversion in chromosome 15. Mitotic cells from Inv268-3c were processed for DNA FISH with a chromosome 15 WCP, and the chromosomal DNA was stained with DAPI. Rearrangements involving chromosome 15 are indicated with arrows, and non-rearranged chromosome 15 s are indicated with asterisks. (TIF) [file pgen.1004923.s007.tif]

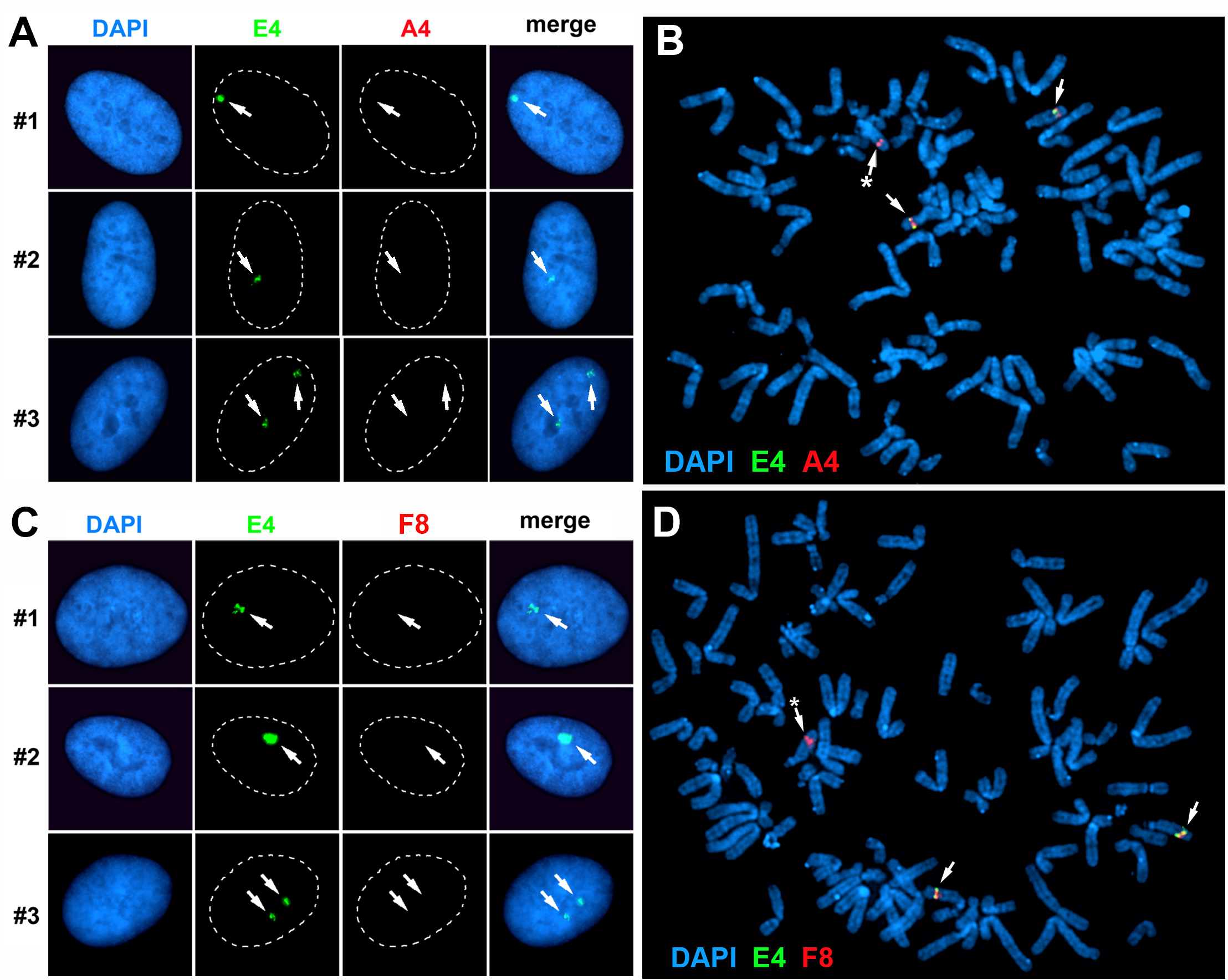

Supplement: S8 Fig — RNA and DNA FISH using fosmid probes A4 and F8 in combination with E4. A) P268 cells were subjected to RNA FISH using the E4 (green) and A4 (red) probes to detect RNA simultaneously. Representative images from three different P268 cells (#1-3) are shown in each panel. The arrows mark the sites of E4 hybridization. B) DNA FISH using E4 (green) and A4 (red) probes to detect DNA simultaneously on metaphase spreads from Δ268F-4t cells, which contain an ∼135 kb deletion that includes the DNA represented by the E4 fosmid but does not delete the A4 fosmid (see Fig. 5A). The arrows mark the sites of hybridization to the E4 plus A4 probes, and the arrow with the asterisk marks the chromosome 15 that only hybridized to the A4 probe. We detected hybridization of the A4 probe to 100% of chromosome 15 s that hybridized to the E4 probe, plus one site of hybridization of the A4 probe to a chromosome 15 without the E4 signal, which allowed us to identify the deleted chromosome 15 s. C) P268 cells were subjected to RNA FISH using the E4 (green) and F8 (red) probes to detect RNA simultaneously. Representative images from three different P268 cells (#1-3) are shown in each panel. The arrows mark the sites of E4 hybridization. D) DNA FISH using E4 (green) and F8 (red) probes to detect DNA simultaneously on metaphase spreads from Δ268F-4t cells, which contain an ∼135 kb deletion that includes the DNA represented by the E4 fosmid but does not delete the F8 fosmid (see Fig. 5A). The arrows mark the sites of hybridization to the E4 plus F8 probes, and the arrow with the asterisk marks the chromosome 15 that only hybridized to the F8 probe. We detected hybridization of the F8 probe to 100% of chromosome 15 s that hybridized to the E4 probe, plus one site of hybridization of the F8 probe to a chromosome 15 without the E4 signal, which allowed us to identify the deleted chromosome 15 s. (TIF) [file pgen.1004923.s008.tif]

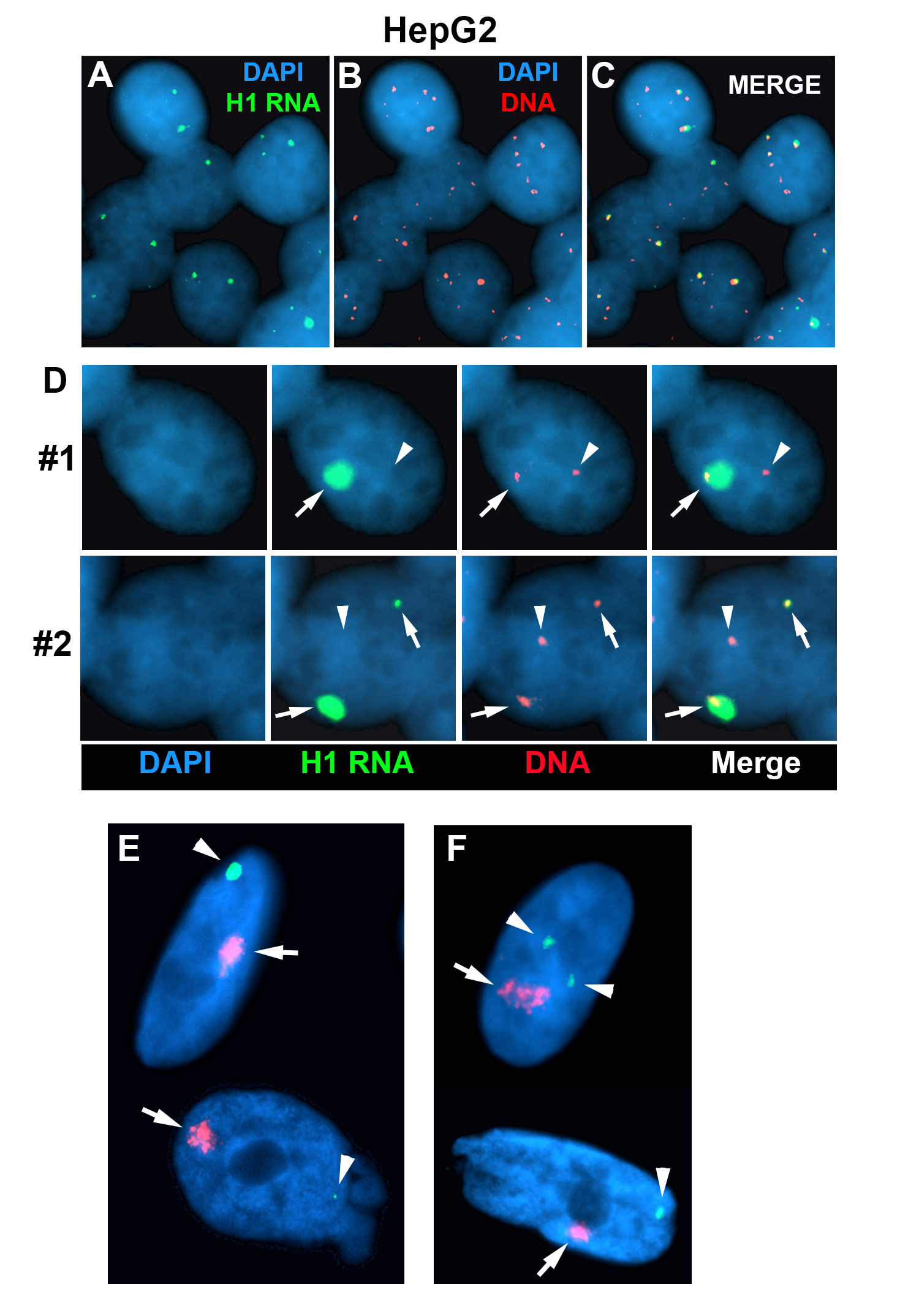

Supplement: S9 Fig — RNA-DNA FISH in HepG2 cells. Human HepG2 cells were processed for RNA-DNA FISH using the H1 probe to detect RNA plus a BAC from the ASAR15 locus to detect DNA. Slides were subjected to RNA FISH using the H1 fosmid (green) to detect RNA. Slides were subsequently processed for DNA FISH using a BAC CTD-2299E17 to detect DNA. The nuclear DNA was stained with DAPI. A–C) A representative field of cells showing the RNA FISH (A), DNA FISH (B) and merged images (C). D) Representative cells (#1 and #2) with large “clouds” of hybridization are shown. The arrows mark the location of the DNA signals that have a corresponding RNA signal, and the arrowheads mark the location of the DNA signals that lack a corresponding RNA signal. The nuclear DNA was stained with DAPI. E and F). RNA FISH in female HDFs. Human female HDF cells were processed for RNA FISH using the H1 ASAR15 probe (green) in combination with an XIST (red) probe. The arrows mark the large clouds of RNA detected by the XIST probe and the arrowheads mark the variably sized hybridization signals detected by the ASAR15 probe. The DNA was stained with DAPI. (TIF) [file pgen.1004923.s009.tif]

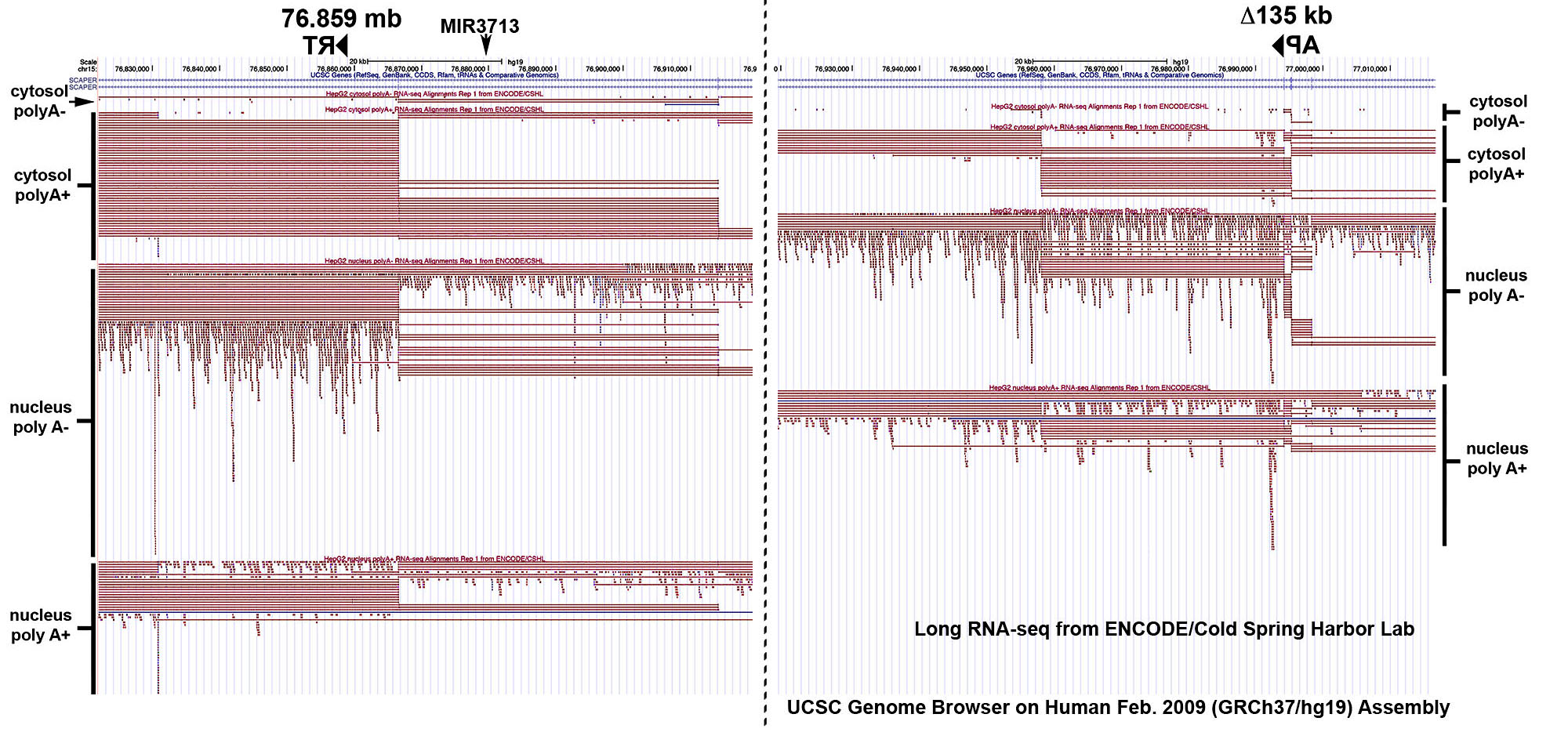

Supplement: S10 Fig — RNAseq data from HepG2 cells using the UCSC Genome Browser. The genomic region between 76,825,000 and 77,015,000 bp of human chromosome 15 (NCBI Build 37/hg19) illustrating SCAPER intron-exon junctions, MIR3713, the location of the original loxP-RT integration site in P268 cells, and the location of the AP-loxP integration site for the ∼135 kb deletion. Two different screenshots, separated by a doted line, show the proximal and distal halves of this region of chromosome 15. Also shown is RNA-seq data, cytosol poly A-, cytosol poly A+, nucleus poly A-, and nucleus poly A+, from the cell line HepG2. The blue tick marks indicate sequence hits from the plus direction, and the red tick marks indicate sequence hits from the minus direction. Note that RNA in the SCPAER introns is enriched in the poly A- fraction in the nucleus, and that there is very little plus strand RNA synthesized, including in the region encoding MIR3713, which is transcribed from the plus strand. (TIF) [file pgen.1004923.s010.tif]

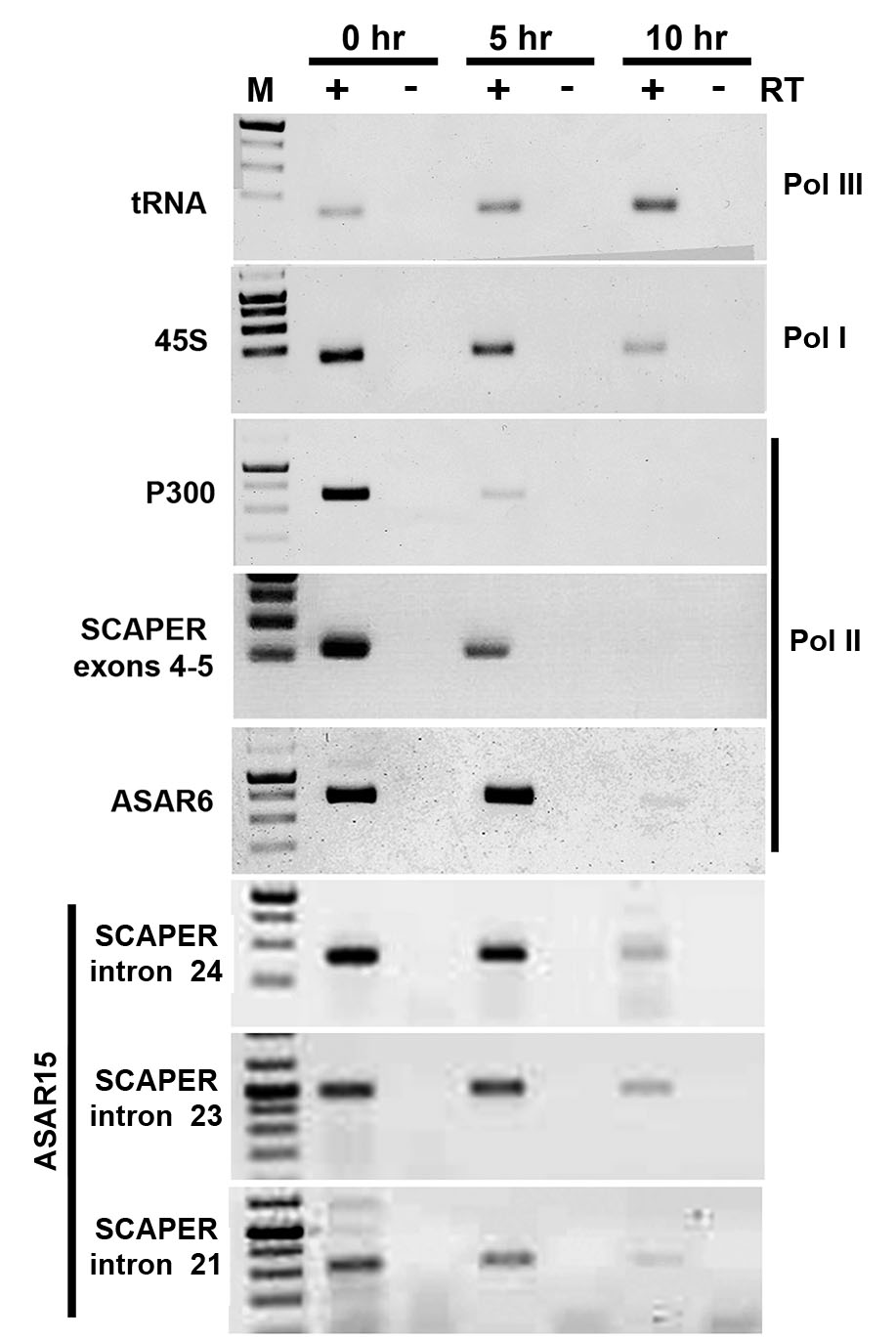

Supplement: S11 Fig — ASAR15 is transcribed by RNA Polymerase II and has a relatively long half-life. Cells were exposed to 20 ug/mL of α-amanitin for 0, 5, and 10 hours. Total RNA was subjected to reverse transcriptase reactions (RT) in the presence (+) or absence (-) of reverse transcriptase followed by semi-quantitative PCR using primers to a tRNA gene (RNA Pol III), 45S RNA (RNA Pol I), P300 cDNA (RNA Pol II), SCAPER cDNA (spanning exons 4 and 5; RNA Pol II) ASAR6 (RNA Pol II), and primers from within SCAPER introns 21 (rs11072602), 23 (rs12916573), and 24 (rs59088060). DNA size ladder was used for size reference (M). (TIF) [file pgen.1004923.s011.tif]
