# Supplementary material for: ASAR15, A cis-Acting Locus that Controls Chromosome-Wide Replication Timing and Stability of Human Chromosome 15
Source: PLoS Genet. 2015 Jan 8;11(1):e1004923. doi: 10.1371/journal.pgen.1004923 (PMC4287527; doi:10.1371/journal.pgen.1004923)
Supplement: S1 Table — Summary of the Cre/loxP-mediated chromosome rearrangements analyzed. The top panel (Deletion Clones) indicates the name of each clone (Δ268-) and the size of the proximal or distal deletions generated from the original loxP-RT integration site in chromosome 15 in P268 cells. The middle panel (Inversion Clones) indicates the name of each clone (Inv268-) and the size of the proximal or distal inversions generated from the original loxP-RT integration site in chromosome 15 in P268 cells. The bottom panel (Aprt transfected Clones) indicates the name of each control P268 clone, generated by transfection of an Aprt expression vector, selection in media containing Azaserine and adenine, and subsequent clonal expansion, analyzed. The number of chromosome rearrangements detected using FISH with a chromosome 15 whole chromosome paint (CHR 15 Paint), and the total number of cells scored are indicated. Also shown are the replication-timing assays (Brdu-WCP and BrdU-BAC) used and whether or not delayed replication timing (DRT) was detected for each clone in each panel. (PDF) [file pgen.1004923.s012.pdf]

Table S1. Summary of the Chromosome analysis of individual clones.

Deletion Clones						
Clone Name	Deletion Size (base pairs)		CHR 15 Paint	Replication Timing		
	Proximal	Distal	Rearrangements*	BrdU-WCP	BrdU-BAC	DRT
Δ268F-6a	>18,125,231		ND	ND	+	-
Δ268F-5a	66,964-126,184Ω		ND	ND	+	-
Δ268F-5d	66,964-126,184Ω		ND	ND	+	-
Δ268F-5m	66,964-126,184Ω		ND	ND	+	-
Δ268F-6b	66,964-126,184#		ND	ND	+	-
Δ268F-6c	66,964-126,184#		ND	ND	+	-
Δ268F-6d	66,964-126,184#		ND	ND	+	-
Δ268-15a		2085	0/100	+	ND	-
Δ268-15c		2085	0/100	+	ND	-
Δ268-18a		124,047	8/100	ND	+	+/-
Δ268-18d		124,047	6/100	+	+	+/-
Δ268-18c		124,047	0/100	ND	ND	N/A
Δ268-18m		124,047	0/100	ND	+	-
Δ268-18n		124,047	1/100	+	+	-
Δ268-4c		135,413	16/50	+	+	+
Δ268-4f		135,413	25/50	+	+	+
Δ268-4k		135,413	29/50	+	+	+
Δ268-4o		135,413	0/100	+	+	+
Δ268-4s		135,413	0/100	+	+	-
Δ268-4t		135,413	0/100	+	+	-
Δ268-4a		161,312	0/100	+	+	+
Δ268-4e		161,312	26/100	+	+	+
Δ268-4g		161,312	6/50	+	+	+
Δ268-4m		161,312	97/100	+	+	+
Δ268-4p		161,312	8/50	+	+	+
Δ268-4s		161,312	0/100	+	+	+
Δ268-18t		255,212	22/100	+	+	+
Δ268-18x		255,212	14/100	+	+	-
Δ268-5c		5,622,178	38/50	ND	+	+
Δ268-5d		5,622,178	96/100	ND	ND	N/A
Δ268-4d		12,833,460	4/50	+	+	+

Inversion Clones						
Clone Name	Inversion Size (base pairs)		CHR 15 Paint	Replication Timing		
	Proximal	Distal	Rearrangements*	Paint/BrdU	BAC/BrdU	DRT
Inv268-3c	76,858,743		38/100	+	ND	+
Inv268-3a		643,704	97/100	ND	ND	N/A
Inv268-6c		784,951	42/100	+	ND	+
Inv268-6e		784,951	6/50	ND	ND	ND

Aprt transfected Clones						
Clone Name			CHR 15 Paint	Replication Timing		
			Rearrangements*	Paint/BrdU	BAC/BrdU	DRT
P268F1			0/100	+	ND	-
P268F2			0/100	+	ND	-
P268F3			0/100	+	ND	-
P268F4			0/100	+	ND	-

* Cells with chromosome 15 rearrangements / total number of metaphase cells scored.
Ω Deletion size was estimated from loss of heterozygosity at SNP rs17363364 and retain of heterozysity at SNP rs17363364.
Deletion size was estimated from loss of heterozygosity at SNP rs17363364 and retain of heterozysity at SNP rs17363364.

ND: not done.
N/A: not applicable.
